# Automated versus physician assignment of cause of death for verbal autopsies: randomized trial of 9374 deaths in 117 villages in India

**DOI:** 10.1186/s12916-019-1353-2

**Published:** 2019-06-27

**Authors:** Prabhat Jha, Dinesh Kumar, Rajesh Dikshit, Atul Budukh, Rehana Begum, Prabha Sati, Patrycja Kolpak, Richard Wen, Shyamsundar J. Raithatha, Utkarsh Shah, Zehang Richard Li, Lukasz Aleksandrowicz, Prakash Shah, Kapila Piyasena, Tyler H. McCormick, Hellen Gelband, Samuel J. Clark

**Affiliations:** 1 Centre for Global Health Research, St Michael’s Hospital and Dalla Lana School of Public Health, University of Toronto, Toronto, Canada; 2 Department of Community Medicine, Pramukhswami Medical College, Anand, Gujarat India; 3 Centre for Cancer Epidemiology, Tata Memorial Centre, Mumbai, India; 4 London School of Hygiene & Tropical Medicine, London, UK; 5 Department of Biostatistics, Yale University, New Haven, USA; 6 Department of Statistics, University of Washington, Seattle, USA; 7 Department of Sociology, Ohio State University, Columbus, USA; 8 Department of Sociology, University of Washington, Seattle, USA

**Keywords:** COD classification, Algorithms, Physician coding, Verbal autopsies

## Abstract

**Background:**

Verbal autopsies with physician assignment of cause of death (COD) are commonly used in settings where medical certification of deaths is uncommon. It remains unanswered if automated algorithms can replace physician assignment.

**Methods:**

We randomized verbal autopsy interviews for deaths in 117 villages in rural India to either physician or automated COD assignment. Twenty-four trained lay (non-medical) surveyors applied the allocated method using a laptop-based electronic system. Two of 25 physicians were allocated randomly to independently code the deaths in the physician assignment arm. Six algorithms (Naïve Bayes Classifier (NBC), King-Lu, InSilicoVA, InSilicoVA-NT, InterVA-4, and SmartVA) coded each death in the automated arm. The primary outcome was concordance with the COD distribution in the standard physician-assigned arm. Four thousand six hundred fifty-one (4651) deaths were allocated to physician (standard), and 4723 to automated arms.

**Results:**

The two arms were nearly identical in demographics and key symptom patterns. The average concordances of automated algorithms with the standard were 62%, 56%, and 59% for adult, child, and neonatal deaths, respectively. Automated algorithms showed inconsistent results, even for causes that are relatively easy to identify such as road traffic injuries. Automated algorithms underestimated the number of cancer and suicide deaths in adults and overestimated other injuries in adults and children. Across all ages, average weighted concordance with the standard was 62% (range 79–45%) with the best to worst ranking automated algorithms being InterVA-4, InSilicoVA-NT, InSilicoVA, SmartVA, NBC, and King-Lu. Individual-level sensitivity for causes of adult deaths in the automated arm was low between the algorithms but high between two independent physicians in the physician arm.

**Conclusions:**

While desirable, automated algorithms require further development and rigorous evaluation. Lay reporting of deaths paired with physician COD assignment of verbal autopsies, despite some limitations, remains a practicable method to document the patterns of mortality reliably for unattended deaths.

**Trial registration:**

ClinicalTrials.gov, NCT02810366. Submitted on 11 April 2016.

**Electronic supplementary material:**

The online version of this article (10.1186/s12916-019-1353-2) contains supplementary material, which is available to authorized users.

## Background

Most of the 45 million annual deaths in low- and middle-income countries (LMIC), out of about 55 million worldwide, occur at home without medical attention, with no cause of death (COD) assigned [1]. Reliable COD information is thus lacking for two thirds of the world’s population, with the lowest coverage in Africa and in low-income Asian countries [2]. For the next few decades, only a minority of deaths in LMICs are likely to occur in facilities where it is feasible to register and medically certify a death based on clinical, laboratory, and diagnostic records. A viable alternative to obtain actual (versus modeled) COD information is to adopt representative, nationwide samples of deaths on which verbal autopsies (VA) are conducted [1, 3].

VA relies on lay (non-medical) field staff to conduct structured interviews of living family members of the deceased to document the key symptoms of the illness (or episode) that led to death that includes past medical and treatment history and additional details [4]. Trained physicians then use this information to assign causes [5–7]. Recent VA studies aim to document national-level mortality patterns and incorporate improved processes such as the use of dual, independent physician assignment, strict coding guidelines, and electronic platforms [3, 6, 7]. Well-conducted national VA studies yield timely, robust, and plausible information on the patterns of death for the major causes and have influenced disease control priorities [1, 8, 9].

Physician assignment is the method traditionally used to code VA results to the WHO International Classification of Diseases (ICD-10). Physician assignment has been criticized as being costly and difficult to implement and potentially suffering from reproducibility gaps between two physicians [10, 11]. Hence, we and other research groups have developed automated, computational algorithms to assign COD based on VA interviews [11–18]. Only observational studies have evaluated the two approaches. These observational studies have produced disparate results [11, 13–20]. Thus, here, we report the results of the first-ever randomized comparison of automated versus the more standard physician COD assignment for VAs.

## Methods

### Trial design

The primary trial design focused on establishing the population-level distribution of CODs and their consistency between physician and automated assignment (Additional file 8). The main public health value of VA data is to inform the population distribution of various CODs [1, 5, 7]. Moreover, earlier reviews have found higher performance for automated assignment at the population versus individual level [11, 18].

The original trial design (ClinicalTrials.gov, NCT02810366; see Additional file 1) called for enrolling 6000 deaths in both arms. We were able to enroll 9374 deaths in two states (covering four mostly rural districts) in Western and Northern India: Gujarat (Anand, Kheda) and Punjab (Mansa, Sangrur), respectively. The Pramukhswami Medical College and Tata Memorial Centre institutional ethics committees approved the study for the respective sites. The pilot study of 1215 deaths in Amravati district in rural Maharashtra, also in Western India, established procedures used in the main study [21] and was excluded from the main analyses. However, the inclusion of these pilot deaths did not alter the outcomes (see Additional file 15).

### Participants

We selected 60 villages in Gujarat that are involved in child health research and 57 in Punjab that are involved in cancer registry studies. Twenty-four trained lay surveyors (15 in Gujarat, nine in Punjab) enumerated all households in each village using a custom, electronic, laptop-based data collection system (see Additional file 1). Following enumeration and after obtaining written consent from respondents aged 18 years or older, the surveyors collected demographic information and conducted VAs on all deaths of household members in the preceding 5 years. The pilot study collected VAs at all ages [21], and as in the Million Death Study (MDS) [3, 7], physicians were unable to assign specific causes to many older decedents, resulting in about a fifth of all deaths in people older than 70 years being “ill-defined”. Thus, the main trial enrolled only deaths below age 70. Results for the 1238 deaths above age 70 in the pilot were similar to those at younger ages (data not shown).

### Randomization and trial procedures

We developed electronic VA forms specific to either physician or automated assignment, which the laptop randomly allocated to each death. The laptop applied balanced randomization to allocate equal numbers to each arm in each village. The surveyor was blinded to assignment and had no influence on the allocation. The software allocated to the relevant arm only after completing demographic questions common to both trial arms. Electronic forms were based on the WHO 2014 standard VA instrument [4]. Completion of all questions, including negative symptoms, was mandatory in each arm. The main difference in the forms between the arms was the inclusion of a narrative of the symptoms and events leading up to death at the end of the interview in the physician assignment arm. Quality control procedures included 10% randomly selected re-interviews by an independent second team member (blinded to the original interview, with five randomly selected questions within each re-interview) (Additional file 19). As well, we audio-recorded the narrative, and the central staff reviewed each surveyor’s narratives once a week and provided feedback.

COD for the automated assignment arm used each of six contemporary algorithms: Naïve Bayes Classifier (NBC) [14], King-Lu [15], SmartVA [13], InSilicoVA [11], InSilicoVA-NT [16], and InterVA-4 [12]. Using a VA training dataset, NBC calculates the conditional probabilities of observing a symptom given a particular COD and uses Bayes’ rule with these probabilities to predict a likely COD. The King-Lu method calculates the symptom and COD distributions in a VA training dataset and uses these to predict the COD distribution for a new set of deaths [15]. The Tariff 2.0 algorithm, made available for use via the openly available SmartVA application, uses training data to calculate tariffs that express the strength of association between symptoms and CODs and applies these through a summing and ranking procedure to identify a COD [19]. InSilicoVA uses a hierarchical Bayesian framework to determine likely CODs with the naïve Bayes calculation as a component. This algorithm also estimates the uncertainty of observing a COD both for an individual death and within the distribution of deaths across the population [11]. InterVA-4 does not need a VA training dataset because it uses clinical expert-defined conditional probabilities of observing each symptom given a particular COD and uses a product of these (related to Bayes’ rule) to determine the likely COD [20]. The InterVA-4 conditional probabilities are also available in the implementation of InSilicoVA. When InSilicoVA uses the InterVA-4 conditional probabilities, we note that fact by labeling the algorithm “InSilicoVA-NT.” Additional file 2 describes each algorithm and details of its application. We used the Population Health Metrics Research Consortium (PHMRC) dataset to “train” NBC, King-Lu, and InSilicoVA; SmartVA is pre-trained on this dataset. This dataset consists of 12,542 health facility deaths at all ages from six sites in Mexico, the Philippines, Tanzania, and two states of India (4552 deaths). It includes a completed VA along with a cause for each death, based on clinical and laboratory information from the health facility [13]. InSilicoVA and InSilicoVA-NT differ only in using symptom cause information of the PHMRC data and of InterVA-4, respectively. None of the algorithms except SmartVA uses the narrative section of the VA interview. SmartVA uses word counts derived from the narrative.

**Fig. 1:**
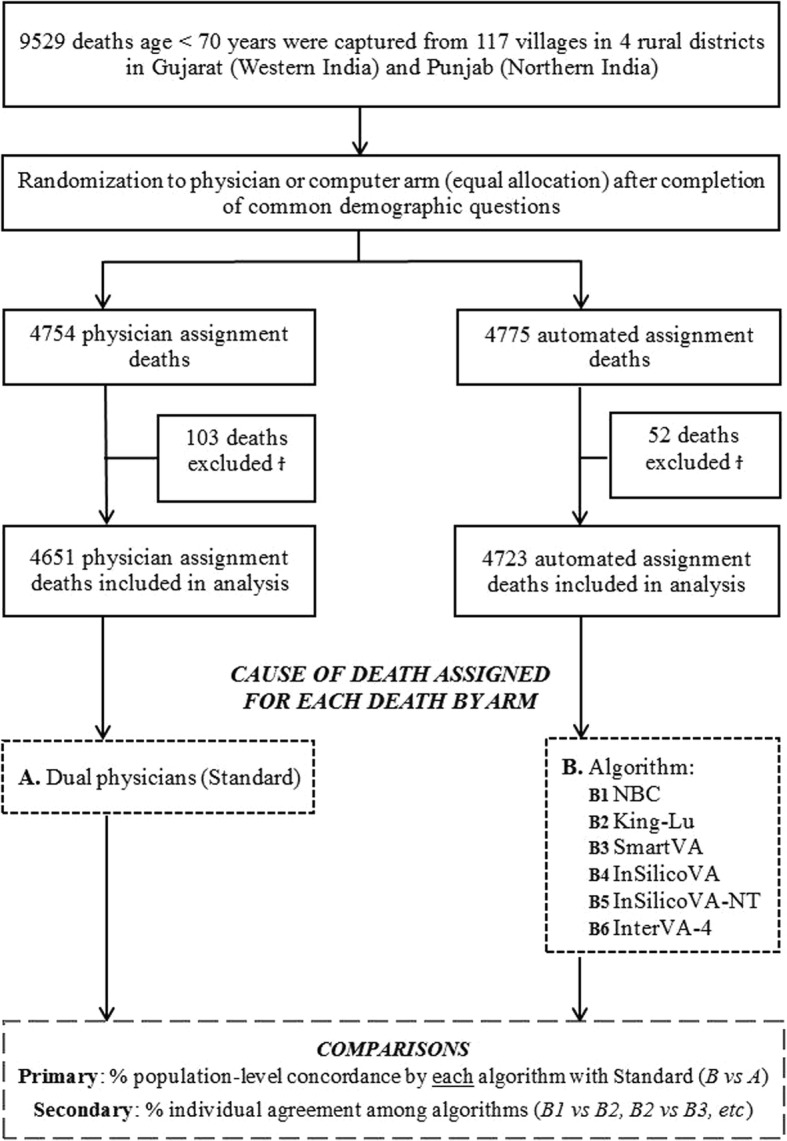
Flow diagram for the 9529 deaths in 117 mainly rural villages randomly allocated to either physician or computer COD assignment of verbal autopsies and analytic design. ^Ϯ^The following deaths were excluded for the physician and automated assignment arms, respectively: 9 and 5 refused consent after the randomization 83 and 39 were unable to provide consent (as the respondent was determined to be < 18 years), and 7 and 8 were test records from field training by surveyors. As well, 4 stillborn deaths were excluded in the physician assignment arm

We used the same procedures as the Indian MDS to assign COD in the standard arm [3, 7, 9]. Records were allocated randomly (based on the ability to read the local language) to two of 25 trained physicians who independently assigned an ICD-10 code as the underlying cause for each death (Fig. 1). Physicians were aware that their coding would undergo anonymous review by another physician. Reconciliation of differences between the two, and, if needed, adjudication by a senior physician, followed. Quadruple physician assignment (i.e., two panels of two physicians each) in the pilot [21] yielded similar results to dual assignment (data not shown); thus, we used dual assignment in the main study. Separately, two physicians (PS, KP) independently classified the deaths in the automated assignment arm to ICD-10, with a senior physician (RB) resolving differences. All physicians were blind to results for either trial arm.

### Statistical analyses

The primary trial outcome was population-level concordance (hereafter “concordance”) that computes the similarity between the COD frequency distributions between the automated arm and the COD frequency distributions in the physician arm (“B vs A” in Fig. 1; see Additional file 3). One hundred percent concordance would mean identical COD *distribution* in the two arms (although not necessarily that each death was coded identically). We grouped ICD-10 codes into 18 cause categories using the MDS classification for adults (12–69 years old), ten for children (28 days to 11 years), and six for neonates (0–27 days; see Additional file 5). All algorithms and physician coding adhered to these cause categories so as to ensure comparability (see Additional file 7). The secondary outcome was individual-level sensitivity (defined as the number of deaths assigned the same COD divided by the total number of deaths) among the five algorithms in the automated arm (“B1” vs “B2”, “B2” vs “B3” and so on in Fig. 1; see Additional file 3). We excluded King-Lu as it does not generate individual CODs [15]. Both measures have been used in past non-randomized studies, and population concordance is identical (see Additional file 3) to the “cause-specific mortality fraction accuracy” used earlier [11, 13, 18].

Based on a significance value of 5% and 80% power, the approximately 4300 adult deaths, 200 child deaths, and 150 neonatal deaths in each arm permitted us to measure concordance up to 97%, 87%, and 84%, respectively, of the algorithms with the standard COD distribution for the relevant age group. We used R for analyses.

## Results

Deaths occurred a mean of 3.2 years before the survey, with results similar for deaths just in the last year (data not shown). In the main trial, we identified 9529 eligible deaths below age 70 across the study sites (Fig. 1). We excluded 155 deaths (103 physician arm, 52 automated arm) mostly due to finding that the respondent was below age 18 years after randomization began (and thus unable to provide consent) or because of other administrative issues (see Fig. 1 footnote). This left 4651 physician-assigned and 4723 automated-assigned deaths for analyses. Random allocation succeeded, as both arms were nearly identical in distributions of location, age, sex, education level, and other demographic features and in the key symptom patterns of the deceased (Table 1; see Additional file 6). Two symptoms captured on a checklist, namely fever and jaundice, differed in the two arms for children and neonates but not for adults, likely representing the play of chance from smaller numbers.

**Table 1:** Baseline characteristics of deaths by study group

	Overall (*n* = 9374)	Standard (physician assignment) (*n* = 4651)	Automated assignment (*n* = 4723)
Study sites			
Gujarat	5174 (55%)	2562 (55%)	2612 (55%)
Punjab	4200 (45%)	2089 (45%)	2111 (45%)
Age groups			
Adult (12–69 years)	8704 (93%)	4311 (93%)	4393 (93%)
Child (28 days to 11 years)	403 (4%)	190 (4%)	213 (5%)
Neonate (0–27 days)	267 (3%)	150 (3%)	117 (2%)
Sex of the deceased*			
Male	6229 (66%)	3086 (66%)	3143 (67%)
Female	3143 (34%)	1564 (34%)	1579 (33%)
Deceased’s education level*			
No formal education	4623 (49%)	2317 (50%)	2306 (49%)
1–9 years	2591 (28%)	1252 (27%)	1339 (28%)
10+ years	1189 (13%)	596 (13%)	593 (13%)
Not applicable as ≤ 5 years	570 (6%)	292 (6%)	278 (6%)
Deceased’s type of house*			
Semi-solid/thatched	7442 (79%)	3702 (79%)	3740 (79%)
Solid	1855 (20%)	915 (20%)	940 (20%)
Location of death*			
Home	6558 (70%)	3233 (70%)	3325 (70%)
Facility	1610 (17%)	812 (17%)	798 (17%)
Other	1190 (13%)	599 (13%)	591 (13%)
Adult key symptoms			
Fever	2834 (33%)	1400 (32%)	1434 (33%)
Breathlessness	2171 (25%)	998 (23%)	1173 (27%)
Chest pain	1896 (22%)	848 (20%)	1048 (24%)
Cough	1798 (21%)	847 (20%)	951 (22%)
Weight loss	1688 (19%)	710 (16%)	978 (22%)
Injury	1554 (18%)	832 (19%)	722 (16%)
Paralysis/stroke	685 (8%)	294 (7%)	391 (9%)
Diarrhea	677 (8%)	299 (7%)	378 (9%)
Jaundice	412 (5%)	191 (4%)	221 (5%)
Child key symptoms			
Fever	179 (44%)	95 (50%)	84 (39%)
Diarrhea	76 (19%)	36 (19%)	40 (19%)
Jaundice	58 (14%)	40 (21%)	18 (8%)
Injury	61 (15%)	24 (13%)	37 (17%)
Cough	57 (14%)	30 (16%)	27 (13%)
Neonate key symptoms			
Breathing problems	60 (23%)	31 (21%)	29 (25%)
Fever	36 (13%)	15 (10%)	21 (18%)
Jaundice	36 (13%)	29 (19%)	7 (6%)
Injury	3 (1%)	1 (1%)	2 (2%)

Data are in (%). Key symptoms refer to a subset of symptoms of each age group that are essential to distinguish various CODs

*Every effort was made to collect data; however, deaths with missing data for sex were 1 (0%) and 1 (0%), for education level were 194 (4%) and 207 (4%), for type of house were 34 (1%) and 43 (1%), and for location of death were 7 (0%) and 9 (0%), for physician and computer assignment study groups, respectively

Using the four-country PHMRC data to train the relevant algorithms, the average concordances with the standard physician-assigned arm were 62%, 56%, and 59% for adults, children, and neonates, respectively (Table 2). InterVA-4 achieved the highest concordance for adults (80%). InterVA-4 and InSilicoVA-NT had the highest concordance for children (66% each), while InSilicoVA attained the highest concordance for neonates (80%). King-Lu had the lowest concordance for adults (44%), and SmartVA the lowest concordance for children (36%) and neonates (27%). Training the algorithms on the India-only subset of data yielded similar results (see Additional file 10).

**Table 2:** Percent population-level concordance in cause of death distribution between automated assignment and standard (physician assignment) verbal autopsies, by algorithms and age groups

		Require training data				Do not require training data	
Age group	Average (SD)	NBC	King-Lu	SmartVA	InSilicoVA	InSilicoVA-NT	InterVA-4
Adult	62 (15)	50	44	57	66	77	80
Child	56 (11)	51	58	36	60	66	66
Neonate	59 (18)	57	68	27	80	54	65

Average and standard deviation (SD) of the population-level concordance attained for the automated algorithms when using data from all PHMRC sites as the training data. The concordance compares the cause of death distributions generated by each algorithm on the 4723 deaths in the automated arm (4393 adult, 213 child, and 117 neonatal deaths) to the distribution on the 4651 standard physician-coded deaths (4311 adult, 190 child, and 150 neonatal deaths). When only the Indian sites were used as the training data, the concordance for NBC, King-Lu, and InSilicoVA was 37, 57, and 68 for adult, 48, 59, and 66 for child, and 23, 76, and 80 for neonatal deaths, respectively. The results were similar if we excluded “ill-defined” deaths (see Additional file 1). InSilicoVA-NT and InterVA-4 do not require training data, whereas SmartVA was pre-trained on the PHMRC data; hence, the percent concordance generated by these algorithms is unchanged when changing the training dataset. Dual physician review of the automated assignment arm generated the population-level concordance of 84, 82, and 91 for adults, child, and neonate age groups, respectively (see Additional file 10)

**Fig. 2:**
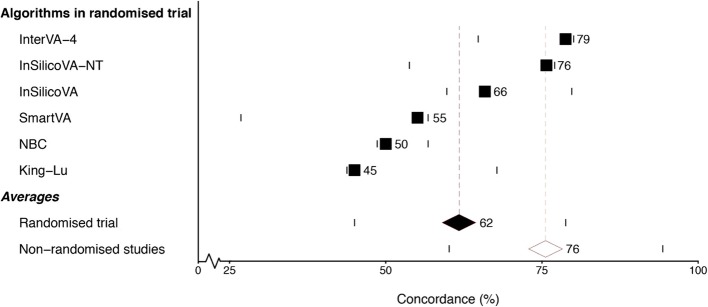
Average population-level concordance (%) of algorithms with standard (physician-assigned) in a randomized trial and the average population-level concordance in earlier non-randomized studies. 100% concordance would indicate complete agreement with the standard. The horizontal bars indicate the range of the mean concordance estimates (weighted by sample size) in each study

Across the three age groups, the overall weighted ranking of best to worst concordance of automated to the physician assignment standard was InterVA-4, InSilicoVA-NT, InSilicoVA, SmartVA, NBC, and King-Lu (Fig. 2). The average concordance of all algorithms was 62% (SD 14%, range 79 to 45%), which was well below the 76% (SD 10%, range 95 to 60%) reported in previous non-randomized studies (which used physician coding or clinical records as standards; see Additional file 9).

Ischemic heart disease was the most frequent cause of adult death based on dual physician coding, accounting for 17% of deaths (Table 3; see Additional file 10). The closest match from algorithms was 13% from InSilicoVA. For the other algorithms, ischemic heart disease accounted for 12% of deaths using InSilicoVA-NT, 8% for King-Lu, 8% for InterVA-4, 5% for NBC, and 4% for SmartVA. Table 3 provides the results for the major causes of adult deaths. Additional files 11 and 12 provide additional details for all age groups. The proportions of specific causes of death assigned by the algorithms versus the standard were quite variable, including the more obvious causes, namely road traffic injury (RTI), cancer, or suicide. We defined obvious causes as those known from independent evidence to be reliably classified when compared to cancer registry [6] or medically certified hospital deaths [22, 23]. For example, among adults, RTI constituted 6% (274/4311 deaths in the standard), of which two physicians agreed on initial diagnosis in 90% (246/274) of cases. Dual independent physician review of the deaths in the automated arm also yielded 6% of adult deaths from RTI (246/4393 deaths in this arm). By contrast, the average of all algorithms predicted RTI as 11% (491/4393) of adult deaths, of which any two algorithms agreed only on was 59% (288/491). InSilicoVA generated an implausible proportion of 28% of adult deaths from RTI (1230/4393), whereas InSilicoVA-NT generated a more plausible proportion of 7% (307/4393). The prevalence of a history of injury deaths, which includes RTI, was similar in the two arms (Table 1).

**Table 3:** Cause of death counts, proportions, and rankings for adults

Rank	Cause of death	No. of deaths				Standard (physician assigned), 7%	Proportion, % (rank)					
		Standard (physician-assigned)		Mean algorithm estimated deaths	2 or more algorithms agreed		Require training data				Do not require training data	
		Total	Both physicians initially agreed^†^				NBC	King-Lu	SmartVA	InSilicoVA	InSilicoVA-NT	InterVA-4
	Adult (12–69 years)											
1	Ischemic heart disease	737	661	368	506	17.1	5.1 (5)	8.3 (5)	4.0 (8)	13.0 (2)	12.8 (2)	8.0 (5)
2	Cancers*	592	517	287	393	13.7	2.0 (12)	1.6 (10)	8.4 (3)	4.4 (10)	10.6 (4)	12.9 (1)
3	Other noncommunicable diseases	376	273	314	365	8.7	6.1 (4)	0.2 (16)	6.1 (5)	6.6 (6)	17.2 (1)	7.5 (6)
4	Unspecified infections	363	265	242	278	8.4	1.8 (13)	0.7 (15)	1.3 (14)	8.5 (4)	12.4 (3)	9.0 (4)
5	Falls, bites, and other injuries*	328	288	811	401	7.6	50.4 (1)	32.7 (1)	9.3 (2)	4.6 (9)	10.1 (6)	5.8 (9)
6	Tuberculosis	303	252	211	283	7.0	3.9 (7)	1.9 (8)	2.3 (10)	0.2 (16)	10.1 (5)	10.9 (3)
7	Chronic respiratory diseases	296	231	175	235	6.9	1.0 (15)	0.9 (14)	8.1 (4)	3.8 (11)	4.3 (9)	6.2 (8)
8	Road and transport injuries*	274	246	491	288	6.4	6.6 (2)	14.9 (2)	5.6 (6)	27.8 (1)	7.0 (7)	6.4 (7)
9	Stroke	232	196	160	197	5.4	2.2 (11)	3.9 (7)	5.2 (7)	4.8 (8)	0.7 (15)	5.5 (10)
10	Suicide*	208	185	200	109	4.8	3.9 (6)	11.7 (3)	1.8 (11)	5.5 (7)	1.9 (11)	3.1 (13)
11	Liver and alcohol related diseases	137	105	63	67	3.2	0.9 (16)	1.5 (11)	0.5 (16)	2.8 (12)	1.6 (12)	1.4 (14)
12	Other cardiovascular diseases	108	64	165	166	2.5	3.1 (8)	1.2 (12)	1.6 (12)	8.5 (3)	3.7 (10)	4.8 (11)
13	Acute respiratory infections	100	72	127	114	2.3	6.1 (3)	1.1 (13)	0.5 (17)	0.5 (14)	4.8 (8)	4.7 (12)
14	Diarrheal diseases	96	68	139	64	2.2	2.8 (10)	6.3 (6)	1.4 (13)	7.9 (5)	0.4 (17)	0.5 (17)
15	Ill-defined	69	59	366	194	1.6	0.0 (18)	0.0 (18)	39.7 (1)	0.0 (18)	0.0 (18)	11.3 (2)
16	Diabetes mellitus	63	49	64	35	1.5	1.3 (14)	1.7 (9)	3.6 (9)	0.3 (15)	0.9 (13)	1.1 (15)
17	Maternal conditions	25	21	121	29	0.6	3.0 (9)	11.4 (4)	0.8 (15)	0.6 (13)	0.6 (16)	0.4 (18)
18	Nutritional deficiencies	4	3	10	4	0.1	0.0 (17)	0.0 (17)	0.0 (18)	0.0 (17)	0.8 (14)	0.6 (16)
	Concordance						50	44	57	66	77	80

*More obvious diagnoses. The order of injuries in this category, from highest to lowest number of deaths, is falls, other injuries, and bites

^†^Percentage of agreement between both physicians at the initial stage of ICD coding, where both physicians assigned the same cause of death for the deceased record. The overall physician initial agreement for adult was 83%; while 18% required a third physician to arbitrate (adjudication)

Compared to the standard, algorithms underestimated cancer and suicide deaths in adults and overestimated other injuries in adults and children. For the above obvious causes, InSilicoVA-NT and InterVA-4 showed the best concordance with the standard. Ill-defined causes were notably higher with algorithms versus physician coding, especially for SmartVA.

We had sufficient numbers to compare individual-level sensitivity on causes only for the 4393 adult deaths in the automated arm (Table 4). The sensitivity for the ten two-way combinations of the five algorithms averaged 30% (SD 16), ranging from 67% between InSilicoVA-NT and InterVA-4 and 18% between InSilicoVA and NBC. By contrast, among adult deaths in the physician arm, 83% (3555/4311) of the 25 randomly assigned physicians agreed initially on the COD. At the population level, the six algorithms showed better concordance of 50% (range 76–36%; see Additional file 13).

**Table 4:** Individual-level sensitivity in the cause of death assignment predicted by different algorithms for adult deaths (12–69 years) in the computer assignment arm (*n* = 4393)

Comparator listed below	SmartVA	InSilicoVA	ISilicoVA-NT	InterVA-4
NBC	22	23	19	18
SmartVA	*	21	38	40
InSilicoVA		*	22	25
InSilicoVA-NT			*	67

Average for the five algorithms: 30 (SD 16). King-Lu only produces population-level results and, thus, was not included. Individual-level sensitivity calculates each algorithm combination (i.e., NBC against SmartVA and SmartVA against NBC). Dual physician review of computer assignment arm produced the following individual-level sensitivity for each computer algorithm: NBC 22; SmartVA 47; InSilicoVA 31; InSilicoVA-NT 51; and InterVA-4 53

*Not applicable

## Discussion

Our randomized trial compared COD assignment by six current automated algorithms to physician assignment, and avoided the inherent problems in observational comparisons of algorithms including the fact that they were trained and tested on differing datasets [18, 24]. The trial adopted rigorous quality control in training, data collection, and coding that yielded high-quality data in both trial arms. Physicians allocated records randomly within their arm, and the physician and automated arms were well-balanced in the overall distribution of the key symptoms predicting COD. We randomized about 50% more deaths than planned originally and had sufficient statistical power to detect high concordance for deaths in each age group. The six algorithms varied widely in their concordance with the standard even for causes that by common sense are easy to identify, such as injury, cancers, or suicide. The range of concordance in our trial overlaps with that in observational studies. However, the veracity of the randomized results is far greater. No one algorithm consistently performed better than the others, with variation in specific diseases. Hence, claims of superiority of any one algorithm [13] carry little scientific credibility.

Physician assignment of COD is the global standard for medical certification of cause of death [4]. Inevitably, the quality of information in VA will be lower than from medically certified deaths occurring in health facilities. However, VA is quite accurate for deaths in children and among young and middle-aged adults (but is less accurate in deaths in older age) when compared to clinical information in hospitals, death certificates, or cancer registry data [6, 7, 22, 23]. Initial agreement by two physicians on the COD was quite high. Importantly, VAs are valuable precisely in settings lacking facility-based certification. Despite the inherent misclassification, VAs are valuably informative compared to no evidence (which is the most common scenario in most countries) and compared to modeled mortality patterns [1]. Though we used physician assignment as the reference, it would be misleading to claim physicians as a “gold” standard, as none exists [1, 7, 12]. Unattended deaths, by definition, cannot be conclusively categorized, and hospital-based deaths cannot adequately reflect home deaths [1].

Additional comparisons enabled in this trial offer reasonable assurance that the use of lay reporting with dual independent physician assignment yields reliable and comparable COD distributions over time and place. First, physician-assigned deaths used as the standard in the trial were distributed similarly to deaths assigned (also by physicians) in the same geographic areas in the most recent data of the MDS (see Additional file 16). Earlier comparison of a 3% random sample of deaths within the MDS showed similar high reproducibility of 94–92% for adults and children below age 5 years (see Additional file 17) [3, 25]. Non-medical VA reporting by field staff provides comparable results to the (far less practical) approach of physicians interviewing VA respondents (see Additional file 18) [13, 26]. Finally, physician assignment of deaths in the automated arm (done only using the list of symptoms without a narrative) yielded concordance of 82–91% with the standard, better than that for algorithms albeit with some variability for specific conditions like ischemic heart disease (see Additional files 11 and 12).

The inadequate performance of current automated algorithms is likely a result of several complementary factors [1, 3, 7, 27, 28]: (i) the intrinsic limitations of each algorithm; (ii) the fact that the PHMRC dataset appears to be customized mostly to build SmartVA (indeed, InterVA-4 and InSilicoVA-NT, which do not require training, generally performed better than algorithms which did, and SmartVA yielded a surprisingly high proportion of ill-defined deaths in adults versus the proportion reported earlier on the PHMRC data [13]); (iii) the PHMRC hospital-based deaths differ substantially from unattended home-based deaths in the education levels, pathogen distribution, and symptom cause information [11, 16, 24, 29]; and (iv) inadequate quality and size of training and testing data (particularly for children and neonates) that limit the ability for algorithms to generate adequate symptom cause information for COD predictions (see Additional files 4 and 14).

Further development of automated methods is desirable, but requires much larger, randomly selected unattended deaths, with sufficient sample size to test combinations for different causes [16]. Currently, it is not possible to specify a priori which algorithm to use for which specific COD. Theoretical, but as yet impracticable, combinations of algorithms would perform much better than individual algorithms (see Additional file 10). Understanding the microbiological status for bacterial and viral infections and pathophysiological processes (such as cerebral edema for malaria) of childhood deaths is now being supported by the Gates Foundation. This may help improve future verbal autopsy tools (and assignment guidelines) by comparing the sensitivity and specificity of symptoms with biological confirmation, particularly if the sampling includes sufficient numbers of home deaths [28, 30]. Natural language processing on VA narratives has also yielded promising results [3]. Narratives contain valuable information on chronology, care-seeking behavior, and social factors which are difficult to capture in checklist interviews [7].

Our results further suggest that programs planning to use automated assignment should retain local language narratives for dual-physician coding. Our trial requires replication in sub-Saharan Africa, where a much higher prevalence of HIV and malaria would result in different mortality patterns to those seen in India.

Considerations of the financial and opportunity costs of physician coding are secondary to the question of accuracy, but information from this trial suggests that the concerns may be misplaced. The entire cost of field work, data collection, and coding per house was less than US $3 (and US $1 in the MDS) [3, 31]. About two thirds of the costs are for the requisite field interviews. Only about one quarter of costs are for physician assignment [3, 7]. The electronic platform used in this trial (and in the MDS) enables physicians to work part-time, typically during evenings, therefore not diverting them from other clinical or public health duties. This study and the MDS reinforce the need to have a large, geographically distributed number of physician coders, so as to help counter biases of any one physician in coding [7]. Standard panels for physician coding and a central pool of doctors to re-code VAs globally would also boost cross-country comparability [4].

Our trial supports the need to develop simpler, cheaper VA field methods [7]. Paradoxically, the 2016 WHO VA forms have 50% more questions than the 2012 version, reaching 346 questions in the adult form (in part to feed demands made to WHO by algorithm designers). Though the MDS has only 68 questions on the adult form, it yielded comparable COD distribution to the longer trial forms (see Additional files 16 and 17). Shorter forms enable quicker interviews that are more likely to retain respondents’ interest, reduce surveyor time costs, and thus enable larger sample sizes [31]. Simplification and reduction of the questions is a priority, while maintaining the ability to use either physician or automated assignment. Ideally, dual independent physician assignment can improve performance and reduce biases of single coding [11]. However, dual physician assignment may not be practical in all settings. Further research on combinations of single-physician coding and resampled second coding, or indeed combining physician and algorithm coding, is required.

## Conclusions

There is a crucial need for direct (versus modeled) measurement of the causes of death to reliably monitor the United Nations’ goals for 2030 [32]. This would involve rapidly expanding the number of LMICs implementing nationally representative COD studies [1], while strengthening civil death registration in the medium term [2]. VAs are essential in settings where medical certification of deaths is uncommon and likely to be obligatory for decades [1]. Lay reporting of unattended deaths with physician COD assignment is widely practicable. Automated methods remain desirable, and their further development should adopt rigorous designs, including the use of randomized evaluations.

## Additional files

Additional file 1: Study method details. (DOCX 19 kb)

Additional file 2: Implementation of automated assignment algorithms. (DOCX 26 kb)

Additional file 3: Statistical tests for comparison. (DOCX 20 kb)

Additional file 4: Summary of limitations of PHMRC data. (DOCX 22 kb)

Additional file 5: Cause of death categories with corresponding ICD-10 codes. (DOCX 23 kb)

Additional file 6: Geographic and age distribution of deaths below age 70 years used in analyses by study group. (DOCX 20 kb)

Additional file 7: Summary of cause list matches between all algorithm results and the cause of death categories used in this study. (DOCX 19 kb)

Additional file 8: Percent population-level concordance in cause of death distribution between algorithms of the automated assignment deaths, by age groups. (DOCX 20 kb)

Additional file 9: Mean population-level concordance (%) comparison of verbal autopsy methods using non-randomized study data, by algorithm. (DOCX 111 kb)

Additional file 10: Percent proportion of causes of death by age groups and training dataset: comparing physician assignment deaths versus the closest automated assignment proportion of deaths for the same cause. (DOCX 25 kb)

Additional file 11: Cause of death counts, proportions, and rankings for adults with dual physician coding of the automated arm. (DOCX 27 kb)

Additional file 12: Cause of death counts, proportions, and rankings for child and neonate age groups with dual physician coding of the automated arm. (DOCX 27 kb)

Additional file 13: Comparison of population-level concordance in cause of death assignment for adults predicted between different algorithms for the automated assignment arm. (DOCX 19 kb)

Additional file 14: Cohen’s Kappa (confidence intervals) between algorithm predictions for adult automated assignment deaths (*N* = 4393). (DOCX 19 kb)

Additional file 15: Percent population-level concordance in cause of death distribution between automated assignment and standard (physician assignment) verbal autopsies, by algorithms and age groups using all results, including pilot site (1215 additional deaths). (DOCX 20 kb)

Additional file 16: Percent of deaths by cause from the standard (physician assignment) arm compared to deaths (2010–2013) in the Indian Million Deaths Study in the trial state by age groups. (DOCX 286 kb)

Additional file 17: Percent of deaths by cause for adults (12–69 years) and children (0–4 years) in the Indian Million Death Study (2001–2013) compared to deaths in the 3% Million Death Study resample. (DOCX 254 kb)

Additional file 18: Percent of sub-study deaths by cause from lay surveyor versus physician collected adult verbal autopsies. (DOCX 188 kb)

Additional file 19: Original study protocol. (DOCX 21 kb)

## Data Availability

The parameters are included in the Supplementary appendix, and the R code is available freely upon written request to the authors. The PHMRC data are publicly available at http://ghdx.healthdata.org/record/population-health-metrics-research-consortium-gold-standard-verbal-autopsy-data-2005-2011. The dataset for this study will also be available to researchers upon written request to the authors (adhering to data sharing agreements with the relevant institutions).

## Abbreviations

COD: Cause of death
eCI: Empirical confidence interval
ICD: International Classification of Diseases
IHME: Institute for Health Metrics and Evaluation
LMIC: Low- and middle-income country
MDS: Million Death Study
NBC: Naïve Bayes Classifier
PHMRC: Population Health Metrics Research Consortium
RGI: Registrar General of India
RTI: Road traffic accident
SD: Standard deviation
SRS: Sample Registration System
VA: Verbal autopsy
WHO: World Health Organization
