# Supplementary material for: Automated versus physician assignment of cause of death for verbal autopsies: randomized trial of 9374 deaths in 117 villages in India
Source: BMC Med. 2019 Jun 27;17:116. doi: 10.1186/s12916-019-1353-2 (PMC6595581; doi:10.1186/s12916-019-1353-2)
Supplement: Supplementary file 1 — Study method details. (DOCX 19 kb) [file 12916_2019_1353_MOESM1_ESM.docx]

**Additional File 1: Study Method Details**

**Training of data collectors**

The 32 lay, non-medical data collectors (eight in Maharashtra, 15 in Gujarat, and nine in Punjab) hired for this study had at minimum completed 12 years of education, and received extensive training of proper field procedures, including a thorough understanding of the structured questions in the verbal autopsy (VA) instruments and detailed instructions on how to collect a VA narrative, with hands on training to electronically complete the questionnaire and narrative. We adopted a logical flow of questions to focus respondents’ early attention on the most relevant symptoms. For example, an interview for a possible death from tuberculosis, as suggested by the respondent, would begin with questions about cough and fever, and not about diarrhoea symptoms. With the inclusion of the narrative for the physician assignment VA forms, the data collectors were rigorously trained through a five step procedure with the ultimate aim of acquiring a summary of the history of symptoms and events leading to death. This process consisted of obtaining: i) positive symptoms, ii) negative symptoms, iii) exploring positive sub-symptoms (eg. duration of fever), iv) gathering treatment history, and the v) ordering all data into chronological order. After capturing all of this information, interviewers would summarize the history and read it back to the respondent to acquire their confirmation that the summary was correct. After confirmation, the interviewer typed these summaries in the computer in the local language used. This process has been explained elsewhere in greater detail; see Aleksandrowicz et al., 2012 and Gomes et al., 2017 for more information. The data collectors employed had to complete an online VA training course, followed by an 11 day in-person group training session, which included training in the field, and finally test successfully on the training material covered. For both VA forms (whether for physician or automation assignment), an eligibility requirement was established, whereby respondents’ needed to be at least 18 or older to participate. The only other age-related restriction was for the deceased age to be under 70 years. Other than that, the only difference between the two forms (physician vs automated assignment) was the inclusion of a half-paged narrative capturing details of the symptoms and events leading to death in the local language for the physician assignment arm.

**Electronic data collection, retrieval, and storage**

During the data collection phase a survey team comprising of two to four surveyors and an identified team leader visited a sampling unit. The team conducted all data collection efforts in this study electronically using Connoi Smart Book Clamshell SBCL13 laptops with a custom-built software platform. At the end of each day, survey team members synchronized the collected household demography data and VAs on each laptop to a team leader’s laptop using a wireless router that created a local area network in the absence of an Internet connection. In turn, the team leaders synchronized their collected information to encrypted servers daily, with the web-based portal enabling data management follow up and quality assurance. All data were strictly encoded, and only managed by a central data team. Randomization was achieved by limiting the difference between the physician assignment (electronic Verbal Autopsy – eVA) and automated assignment (Extended Symptoms List – ESL) interviews to two on each surveyor laptop. This ensured that the maximum difference between eVA and ESL counts for the overall study (Amravati/Anand/Mansa) would never exceed two for any surveyor in each village.

**Physician coding of verbal autopsies**

Following data collection, we used independent, dual physician cause of death (COD) assignment to ascertain COD for deaths marked for physician assignment, with reconciliation of differences and adjudication by a senior physician if needed. We employed a total of 25 part-time physicians who met the following criteria for this task: fluent knowledge of at least one of the three Indian languages in which this multicentre trial was conducted (Marathi, Gujarati, and/or Punjabi); and having successfully completed rigorous online training in WHO guidelines for assigning the International Classification of Diseases version 10 (ICD-10) codes (WHO, 2008). The software system used in this trial randomly assigned completed physician coding VA forms to two of the 25 employed physicians sorted only by language of VA data collection and physicians’ knowledge of the same. The software removed identifying information of the deceased and respondent and replaced it with a unique identifier before sending the VA record to the physicians. The physicians, however, had information on the state in which the deceased died. The physicians received an email alert when a VA record had been assigned to him/her. The software provided a web interface for the physicians to log into, review the allocated records, and assign an ICD-10 code. This web-interface also provided COD assignment guidelines based on expert criteria (Sinha et al., 2006), and suggested differential diagnoses for major CODs (the top three alternative diagnoses, based on analysis of earlier Indian Million Death Study (Aleksandrowicz et al., 2014) physician disagreements in over 120,000 deaths). It allowed for unrestricted use of more than 2,000 three-digit ICD-10 codes, including a search function to find any specific code, and age and gender checks for incorrect classification (such as cervical cancer in males). If the assigned COD for a VA did not agree between the two physicians the web-interface made the “key words” (symptoms and signs identified by a physician to support their diagnosis) and the diagnosis of the other (anonymous) physician available and gave each physician a chance to revise their COD assignment. If neither physician relented in their original diagnosis, a third more senior physician received the VA records, key words, and diagnoses assigned by the two anonymous physicians in the previous COD assignment rounds, and determined the final ICD-10 COD code. For 70% of the records, physicians were in immediate agreement and no further adjudication was required (data not shown).

An additional form of validation was performed on the deaths captured for the computer COD assignment arm via dual physician review. As described above, the same procedure was conducted on this arm whereby two independent physicians (out of the 25 physicians) assigned an ICD-10 code to each death with anonymous reconciliation and adjudication.

**Study Protocol Registration and Amendments**

This study was registered (ClinicalTrials.gov, NCT02810366) and approved by the Pramukhswami Medical College and Tata Memorial Centre institutional ethics committee. The trial pilot and main trial were intended to be registered prior to the start of the pilot at the International Institute of Population Sciences (IIPS. The pilot began in October 2015. However, there was an administrative delay, and the trial was registered first in April 2016, and updated in June 2016. During the period of October 2015-April 2016, the pilot helped to refine the methods (such as restricting the death range to age 70 years and using double versus quadruple physician coding). The main trial enrolment occurred from June 2016 to Nov 2016 in the main trial sites.

Details on the study are outlined in the original protocol, which can be found on pages 26 onward. The following amendments were made to the protocol a) a total of 10 589 deaths were captured and randomized, which differs from the originally planned sample size of 6000 (an increase in sample size reduced the potential discrepancy as at 5% significance and 80% power, we can detect a 2.5% discrepancy (or 97.5% concordance) with a sample size of 8600, and with a sample size of 3000 we can detect a 4% discrepancy) ; b) the first site, Amravati district in Maharashtra capturing 1215 deaths, was used as a pilot study to test the planned protocol procedures (led by the International Institute for Population Sciences); and c) individual level measure of sensitivity was computed and included in the main text, substituting the originally planned Cohen’s Kappa metric. This substitution occurred due to the fact that both measures produced similar results. Although the Kappa statistic is not reported in the main text, it was measured and included in the Supplementary Tables and Figures section as Web Table 8.

Although the pilot deaths were not incorporated, the results that include these deaths were entirely consistent with the main study results reported in the paper, see Web Table 9.
