# Supplementary material for: Automated versus physician assignment of cause of death for verbal autopsies: randomized trial of 9374 deaths in 117 villages in India
Source: BMC Med. 2019 Jun 27;17:116. doi: 10.1186/s12916-019-1353-2 (PMC6595581; doi:10.1186/s12916-019-1353-2)
Supplement: Supplementary file 2 — Implementation of automated assignment algorithms. (DOCX 26 kb) [file 12916_2019_1353_MOESM2_ESM.docx]

**Additional File 2: Implementation of Automated Assignment Algorithms**

**Automated COD assignment of verbal autopsies**

Automated assignment records were assigned a COD using each of the five leading automated VA classification algorithms independently: naïve Bayes Classifier (NBC) (Miasnikof et al., 2015), King-Lu (King & Lu, 2008), Tariff 2.0 (Serina et al., 2015), InSilicoVA (McCormick et al., 2016) and InterVA-4 (Byass et al., 2012). Using a VA training dataset, NBC calculates the conditional probabilities of observing a symptom given a particular COD, and uses Bayes’ rule with these probabilities to predict a likely COD (Miasnikof et al., 2015). The King-Lu method calculates the symptom and COD distributions in a VA training dataset and uses these to predict the COD distribution for a new set of VAs (King & Lu, 2008). The Tariff 2.0 algorithm, made available for use via the openly available application SmartVA (Institute for Health Metrics and Evaluation [IHME], 2018a), uses training data to calculate tariffs that express the strength of association between symptoms and CODs and applies these through a summing and ranking procedure to identify a COD (Serina et al., 2015). InSilicoVA uses a hierarchical Bayesian framework to determine likely CODs with the naïve Bayes calculation as a component. This algorithm also estimates the uncertainty of observing a COD both for an individual death and within the distribution of deaths across the population (McCormick et al., 2015). InterVA-4 does not need a VA training dataset because it uses clinical-expert-defined conditional probabilities of observing each symptom given a particular COD and uses a product of these (related to Bayes’ rule) to determine the likely COD (Byass et al., 2012). The InterVA-4 conditional probabilities are also available in the implementation of InSilicoVA. When InSilicoVA uses the InterVA-4 conditional probabilities, we note that fact by labelling the algorithm ‘InSilicoVA-NT’.

This section provides details on how each algorithm was implemented to predict causes of deaths for the automated assignment records. The software requirements, setup, and usage examples are provided to demonstrate the methods used in this paper.

## R Setup

R was used as a programming language for various data processing and algorithm tasks. R was used in combination with RStudio to implement the methods and run the algorithms described in this paper. The following steps were taken to install a standard R environment on a machine:

Install R (see https://cran.r-project.org)

Install RStudio (see https://www.rstudio.com)

Open RStudio

Run code in the R console of RStudio

## Naïve Bayes Classifier

Using a training dataset, the naïve Bayes Classifier (NBC) algorithm calculates the probability of assigning causes of death to each case from a set of symptoms assumed to be independent (Miasnikof et al., 2015). Each symptom in the training and testing datasets was coded as 0 or 1, where 0 indicated absence of the symptom and 1 indicated presence of the symptom. The causes of death with the highest probability for each case were assigned as the predicted causes of death. Population Health Metrics Research Consortium (PHMRC) (IHME, 2018b) data was used as the training data, while the computer assignment VA data was the testing data. Due to the difference in questions between the datasets, the training and testing data only contained values corresponding to similar, if not identical, questions found in both age-specific VA questionnaires. Age group-specific computer assignment VA questions mapped to 87 (82%), 45 (73%), and 45 (73%) questions from the PHMRC shortened VA forms for adult, child, and neonate age groups, respectively; since data was available from PHMRC’s long form VA questionnaire, an additional 33, 17, and 24 questions for adult, child, and neonate were mapped, respectively, and included in the final training and testing datasets. The final datasets had 114, 54, and 55 unique symptoms for adult, child, and neonate age groups. The results were mapped to 18 adult, ten child, and six neonate age group-specific cause categories on ICD-10 code as listed in Table 1 of the supplementary tables and figures section. Additional details regarding the mapping of cause categories can be found in Table 3 of the supplementary tables and figures section.

The software used for applying the NBC algorithm in this paper was available online as an R package named *nbc4va* from the Comprehensive R Archive Network (CRAN) (Wen, 2016). Software specifications are provided below:

**Software:** nbc4va

**Version:** 1.0

**Operating System:** Windows, Mac, Linux

**Link:** https://cran.r-project.org/package=nbc4va

The *nbc4va* software was run on a machine with the following specifications:

**Operating System:** Windows 7 (32-bit)

**Processor:** Intel Core i3-3120M (2.5 GHz)

**Memory:** 4 GB

**Storage:** 300 GB HD

**Miscellaneous:** R (version 3.3.2), RStudio (version 1.0.136)

The parameters used for the *nbc4va* software were:

**train:** R Dataframe of training data with symptoms and without causes of death

**test:** R Dataframe of testing data with symptoms and causes of death

**known:** TRUE

The *nbc4va* software (Wen, 2016) was installed on the machine by entering *install.packages("nbc4va")* in an R console. The default parameters were used for the *nbc* function. An example in R is demonstrated below:

# install.packages("nbc4va")

# Load the library and sample data

library(nbc4va)

data(nbc4vaData)

# Split sample data into train and test sets

train <- nbc4vaData[1:50, ]

test <- nbc4vaData[51:100, ]

# Run NBC for results

results <- nbc(train, test)

# Alternatively, a graphical user interface can be used

nbc4vaGUI()

**King-Lu**

The King-Lu algorithm measures the symptom and cause of death distributions in the verbal autopsy (VA) training dataset and uses this to predict the cause of death distribution for a set of new VAs, the “testset” (King & Lu, 2008). Each symptom in the training and testing datasets was coded as 0 or 1, where 0 indicated absence of the symptom and 1 indicated presence of the symptom. The output of the algorithm is a predicted cause of death distribution for the VA records in the testset. PHMRC (IHME, 2018b) data was used for the training data, while the computer coding VA data was the testing data. Due to the difference in questions between the datasets, the training and testing data only contained values corresponding to similar, if not identical, questions found in both age-specific VA questionnaires. Age group-specific computer assignment VA questions mapped to 87 (82%), 45 (73%), and 45 (73%) questions from the PHMRC shortened VA forms for adult, child, and neonate age groups, respectively; since data was available from PHMRC’s long form VA questionnaire, additional 33, 17, and 24 questions for adult, child, and neonate were mapped, respectively, and included in the final training and testing datasets. The final datasets had 114, 54, and 55 unique symptoms for adult, child and neonate age groups. The results were mapped to 18 adult, ten child, and six neonate age group-specific cause categories on ICD-10 code as listed in Table 1 of the supplementary tables and figures section. Additional details regarding the mapping of cause categories can be found in Table 3 of the supplementary tables and figures section.

The software used for applying the King-Lu algorithm in this paper was available online as an R package. Software specifications are provided below:

**Software:** VA

**Version:** 0.9-2.12

**Operating System:** Windows, Mac, Linux

**Link:** https://gking.harvard.edu/va

The King-Lu software was run on a machine with the following specifications:

**Operating System:** Windows 7 (32-bit)

**Processor:** Intel Core i3-3120M (2.5 GHz)

**Memory:** 4 GB

**Storage:** 300 GB HD

**Miscellaneous:** R (version 3.3.2), RStudio (version 1.0.136)

An R demonstration for installation and parameter selection used in this analysis for the *King-Lu* software package is included below:

# install.packages

# Load the libraries

install.packages("quadprog")

install.packages("C:/Users/Downloads/VA_0.9-2.12.tar.gz", type = "source", repos = NULL)

library("quadprog")

library(VA)

library(data.table)

#find optimal number of symptoms

#nsymp.vec over 20 throws an error that you have too many symptoms to test

#neonates

minSymp = 7

maxSymp = 12

#children

minSymp = 7

maxSymp = 18

#adults

minSymp = 12

maxSymp = 20

numSymp <-va.gcv(formula = as.formula(paste(paste(headerNames, collapse="+"), "~cause")), data=list(hospitalData, communityData), nsymp.vec=minSymp:maxSymp, n.subset=300, prob.wt=1, boot.se=FALSE, nboot=1, printit=FALSE, print.reg.size=FALSE)

#run KingLu

result <-va(formula = as.formula(paste(paste(headerNames, collapse="+"), "~cause")), data=list(hospitalData, communityData), nsymp=numSymp$best.nsymp, n.subset=300, prob.wt=1, boot.se=FALSE, printit=TRUE)

**SmartVA**

The SmartVA-Analyze application implements the Tariff 2.0 Method (Serina et al., 2015). Tariff 2.0 was trained on the PHMRC VA database to calculate tariff scores that related to the strength of association between symptoms and CODs (IHME, 2018b). The Tariff 2.0 algorithm uses the tariff scores in a summing and ranking procedure to identify a COD. The algorithm assigns a cause of death category from a target cause list which was developed from the WHO’s estimates of the leading CODs in developing countries in 2004.

The tool requires the input test datasets to be formatted in the exact same way as the PHMRC shortened instrument (which can be accessed here: http://www.healthdata.org/verbal-autopsy/tools), in terms of the ordering of questions, the re-coding of responses, and the heading labels. A total of 246 symptom-related questions (123 out of 182 adult-specific, 79 out of 129 child-specific, and 70 out of 121 neonate-specific questions) were matched to the PHMRC shortened instrument, and reformatted according to tool specifications as the input test dataset. In addition to creating the individual-level and population-level estimates as tables and graphs, the tool generates a warnings file, highlighting any variables that contain illegal values (i.e. values out of range or unexpected, or violating skip patterns and the PHMRC instrument), and setting them to the default value to continue analyzing the input data (IHME, n.d.). All warning files were checked to ensure that the input data would be accurately analyzed by the SmartVA tool, and all check boxes were selected as the data fulfilled the requirements; selections included that the VA dataset had questions pertaining to health care experience (i.e. “Did a doctor ever diagnose HIV/AIDS?”), that malaria is prevalent in India, and that data contained some free text variables. All test inputs had free text included where there was an appropriate match between the computer coding VA data and PHMRC shortened instrument (i.e. notes from health care records). The results were mapped to 18 adult, ten child, and six neonate age group-specific cause categories on ICD-10 code as listed in Table 1 of the supplementary tables and figures section. Additional details regarding the mapping of cause categories can be found in Table 3 of the supplementary tables and figures section.

The SmartVA software specifications are detailed below:

**Software:** SmartVA

**Version:** Smart VA 1.1.1

**Operating System:** Windows

**Link:** http://www.healthdata.org/verbal-autopsy/tools

The SmartVA software was run on a machine with the following specifications:

**Operating System:** Windows 7 (32-bit)

**Processor:** Intel Core i3-4030U (1.90 GHz)

**Memory:** 4 GB

**Storage:** 465 GB HD

The parameters used for the SmartVA software were:

**Data origin (country):** India (IND)

**Malaria region:** check box

**Health Care Experience (HCE) variables**: check box

**Free text variables:** check box

**InSilicoVA and InSilicoVA-NT**

The original InSilicoVA algorithm uses the same symptom and cause lists as InterVA-4, defined in the WHO 2012 standard VA instrument. We denote this version of InSilicoVA as InSilicoVA-NT. InSilicoVA-NT uses the same expert-defined conditional probabilities of a symptom given a cause to fit the model and does not require any training data. When using training data instead of the built-in probabilities, InSilicoVA replaces the InterVA-4 conditional probabilities with similar conditional probabilities recalculated from the PHMRC data set (McCormick et al., 2015). Each symptom in the training and testing datasets was coded as NA, 0, or 1, where NA indicated missing of symptom, 0 indicated absence of the symptom, and 1 indicated presence of the symptom. The causes of death with the highest probability for each case were assigned as the predicted causes of death, and the estimated probability of each cause of death for each death is also provided. The InSilicoVA algorithm also estimates the population cause-specific mortality fractions. Due to the small sample sizes in some experiments, we aggregate the probability of each cause of death for each death to obtain the cause-specific mortality fractions specific to the test data sample. The results using the estimated population cause-specific mortality fractions are very similar in experiments where sample size is moderate. PHMRC data (IHME, 2018b) was used as the training data, while the computer coding VA data was the testing data. Due to the difference in questions between the datasets, the training and testing data only contained values corresponding to similar, if not identical, questions found in both age-specific VA questionnaires. Age group-specific computer assignment VA questions mapped to 87 (82%), 45 (73%), and 45 (73%) questions from the PHMRC shortened VA forms for adult, child, and neonate age groups, respectively; since data was available from PHMRC’s long form VA questionnaire, an additional 33, 17, and 24 questions for adult, child, and neonate were mapped, respectively, and included in the final training and testing datasets. The final datasets had 114, 54, and 55 unique symptoms for adult, child, and neonate age groups. The results were mapped to 18 adult, ten child, and six neonate age group-specific cause of death categories on the labels associated with ICD-10 codes listed in Table 1 of the supplementary tables and figures section. Additional details regarding the mapping of cause categories can be found in Table 3 of the supplementary tables and figures section.

The main software used for applying the InSilicoVA algorithm in this paper was available online as an R package named *InSilicoVA* (Li, 2017). Another R package named *openVA* (Li, 2017) was used to call the algorithm functions and organize the results. Both packages can be obtained from the Comprehensive R Archive Network (CRAN). Software specifications are provided below:

**Software:** InSilicoVA

**Version:** 1.1.5

**Operating System:** Windows, Mac, Linux

**Link:** <https://cran.r-project.org/package=InSilicoVA>

**Software:** openVA

**Version:** 1.0.3

**Operation System:** Windows, Mac, Linux

**Link:** <https://cran.r-project.org/package=openVA>

The *InSilicoVA* and *openVA* software were run on a machine with the following specifications:

**Operating System:** macOS High Sierra

**Processor:** Intel Core i7 (2.6 GHz)

**Memory:** 8 GB

**Storage:** 250 GB SSD

**Miscellaneous:** R (version 3.4.3), Microsoft R Open (version 3.4.3)

The parameters used for the *openVA* software were:

**train:** R Dataframe of training data with symptoms and without causes of death

**test:** R Dataframe of testing data with symptoms and causes of death

**Nsim:** Integer number of iterations to perform resampling. Set to 10,000 in experiments.

**auto.length:** Logical indicator for automatically increasing Nsim. Set to FALSE.

**Causes.table:** List of causes of death

**Other tuning parameters:** Set to package default values.

Both packages were installed on the machine by entering *install.packages("openVA")* in an R console. An example in R is demonstrated below:

# install.packages("openVA")

# Load the library and sample data

library(openVA)

data(RandomVA3)

# Split sample data into train and test sets

train <- RandomVA3 [1:200, ]

test <- RandomVA3 [201:400, ]

# Run InSilicoVA for results

fit <- codeVA(data = test, data.type = "customize", model = "InSilicoVA",

data.train = train, causes.train = "cause",

Nsim=1000, auto.length = FALSE)

## InterVA-4

The InterVA-4 algorithm identified a propensity for 62 pre-defined causes of death for each case using clinical-expert-defined conditional probabilities of a symptom given a cause in an adaptation of Bayes’ rule that removes factors having to do with symptoms that are not present (Byass et al., 2012; McCormick et al., 2016). The symptom and cause lists used by InterVA-4 are pre-defined by the 2012 World Health Organization (WHO) Verbal Autopsy (VA) instrument and causes of death are compatible with the International Classification of Diseases version 10 (ICD-10). It requires an input dataset with values from 245 questions coded as ‘Y’ for yes, blank for no and ‘.’ for unknown responses; the computer assignment VA data was mapped to 213 questions, containing ‘.’ responses for the remaining unmatched questions. The COD labels in the output were matched to the broad cause of death categories found in Table 1 of the supplementary tables and figures section. Additional details regarding the mapping of cause categories can be found in Table 3 of the supplementary tables and figures section.

The InterVA-4 software specifications are detailed below:

**Software:** InterVA-4

**Version:** 4.03 2016-01-12

**Operating System:** Windows

**Link:** http://www.interva.net/

The InterVA-4 software was run on a machine with the following specifications:

**Operating System:** Windows 7 (32-bit)

**Processor:** Intel Core i3-3120M (2.5 GHz)

**Memory:** 4 GB

**Storage:** 300 GB HD

**Miscellaneous:** R (version 3.3.2), RStudio (version 1.0.136)

The parameters used for the InterVA-4 software were:

**Output log name:** valog.txt

**Output data name:** memvars.csv

**Batch file name:** batchin.csv

**Reading or delimited:** D

**Malaria Prevalence:** High

**HIV/AIDS Prevalence:** Low

The original InterVA-4 software was installed on the machine by downloading a zip file from the official website (http://www.interva.net/) under the products section, extracting the zip file into a folder, and running the extracted Windows batch file (interva4_03.bat) to generate a graphical user interface. The user interface required user input of parameters for the log file, data file, batch file (input dataset to be predicted on), type of output (reading or delimited), malaria prevalence, and HIV (Human Immunodeficiency Virus) prevalence. The parameters used were *log=valog.txt/data=memvars.csv/batch=batchin.csv* (the defaults for the input and output files), *type=D* (type of output set to delimited), *HIV=h* (high level of HIV prevalence), and *malaria=l* (low level of malaria prevalence), where *h* means high and *l* means low (given a possibility of three choices: very low, low, and high). After producing the output files with the *InterVA-4* software, the 1^st^ column of the delimited log file (valog.txt) was used to identify each case, and the 8^th^ column was used as the predicted causes of death output.
