# Supplementary material for: Automated versus physician assignment of cause of death for verbal autopsies: randomized trial of 9374 deaths in 117 villages in India
Source: BMC Med. 2019 Jun 27;17:116. doi: 10.1186/s12916-019-1353-2 (PMC6595581; doi:10.1186/s12916-019-1353-2)
Supplement: Supplementary file 3 — Statistical tests for comparison. (DOCX 20 kb) [file 12916_2019_1353_MOESM3_ESM.docx]

**Additional File 3: Statistical Tests for Comparisons**

Computing Population-Level Concordance

We define population-level concordance identically to the cause-specific mortality fraction (CSMF) accuracy metric published earlier (Leitao et al., 2014; Murray et al., 2014). This metric enables the assessment of the overall performance of an algorithm for predicting the cause of death (COD) distributions across all causes. The closer the value is to 1, the more accurate the predictions. Here, we compared the COD distributions generated by each algorithm in the automated assignment arm against the *true* standard COD distributions (which we took as that coded by two of 25 physicians with anonymous reconciliation and adjudication in the physician assignment arm). It is represented in the following formula:

$$CSMF Accuracy=1-\frac{\sum_{j=1}^{k} \left( {CSMF}_{j}^{true}- {CSMF}_{j}^{pred} \right)}{2 (1-Minimum\left( {CSMF}_{j}^{true} \right))}$$

**CSMF^true^** = Physician assigned COD distribution proportions of the Standard Physician assignment arm (“A” in Figure 1; Standard)

**CSMF^pred^** = Algorithm assigned COD distribution proportions of the Automated assignment arm (“B1 to B6” in Figure 1; Algorithms)

$\sum_{\boldsymbol{j=1}}^{\boldsymbol{k}} \left( \boldsymbol{CSMF}_{\boldsymbol{j}}^{\boldsymbol{true}}\boldsymbol{-}\boldsymbol{CSMF}_{\boldsymbol{j}}^{\boldsymbol{pred}} \right)$ = Sum of the absolute differences between the true and predicted COD distribution proportions

As a worked example we show the actual results for three adult conditions:

| Cause | Standard (physician assigned) **(CSMF^true^) (A)** | NBC prediction **(CSMF^pred^) (B)** | Absolute Difference  **(A-B)** |
| --- | --- | --- | --- |
| Ischemic heart disease | 0.170958 | 0.050534942 | 0.120423 |
| Tuberculosis | 0.070285 | 0.038925563 | 0.031359753 |
| Suicide | 0.048249 | 0.039153198 | 0.009095 |
| + 13 more conditions | … | … | … |
| **Σ (CSMF^true^ - CSMF^pred^)** | | | **1.006279** |
| **Minimum CSMF^true^** | | | **0.000928** |

$$CSMF Accuracy =1-\frac{1.006279}{2 (1-0.000928)}$$

$$= 1-\frac{1.006279}{1.998144282}$$

$$=50\% (0.49639)$$

Computing Sensitivity at the Individual-Level

We used a simple version of the sensitivity for individual-level deaths among the five algorithms (as King Lu method calculates only population level results). This is simply the number of deaths assigned the same COD divided by the total number of deaths. These yielded similar results to the Chance Corrected Concordance, which computes a concordance score that adjusts for chance (CCC; Murray et al., 2014). For example the CCC results for 4393 adult deaths in computer COD assignment arm averaged 35% for the ten comparisons (any two of the five automated assignment algorithms ranging from 73% between InSilicoVA-NT and InterVA-4 and 37% between InSilicoVA and NBC; data not shown)) similar to the average sensitivity of 30% for the ten comparisons in Table 3.

Another individual level measure of agreement (also known as a measure for interrater reliability) used was Cohen’s Kappa statistic (κ). It measures the proportion of agreement, similar to sensitivity, while accounting for chance. It produces a value between -1 to 1, where a score below 0 denotes that the agreement was poorer than agreement made by chance, a 0 score represents agreement was equivalent to agreement made by chance, a score above 0 indicates the agreement was better than agreement made by chance, and a score of 1 means perfect agreement (Fleiss and Cohen, 1973). It is represented by the following formula:

$$\kappa=\frac{n_{a}- n_{e}}{n- n_{e}}$$

n_a_ = number of deaths where algorithm 1 and algorithm 2 assigned the same COD

n_e_ = number of deaths that algorithm 1 and algorithm 2 are expected to assign the same COD based on chance; for each COD category, the CSMFs from algorithms 1 and 2 are multiplied together to create a new rate that represents the rate that both algorithms would predict the COD. This resultant rate is then multiplied against the total amount of deaths studied, and later summed to obtain the expected amount of deaths that both algorithms would classify identically due to chance. This is shown in the following equation:

n_e_ = Σ ((CSMFs_algorithm 1_ * CSMFs_algorithm 2_) * n)

n = total number of deaths

Kappa scores and the respective confidence intervals were computed using the rel package (version 1.3.1, available on CRAN) in R (version 3.4.2) which is publicly available. For each age group, all individual-level results (COD predictions made by each algorithm) were combined (every row represents one death with every algorithm COD prediction), and Kappa scores with confidence intervals were generated using the following syntax (where adult represents the combined results dataset with all algorithm predictions for the adult deaths):

#Install package

install.packages(“rel”)

#Run package

library(rel)

#Generate Kappas:

ckap(adult[,c(3,4)], weight = “unweighted”, std.err = “Cohen”)
