# Supplementary material for: Automated versus physician assignment of cause of death for verbal autopsies: randomized trial of 9374 deaths in 117 villages in India
Source: BMC Med. 2019 Jun 27;17:116. doi: 10.1186/s12916-019-1353-2 (PMC6595581; doi:10.1186/s12916-019-1353-2)
Supplement: Supplementary file 4 — Summary of limitations of PHMRC data. (DOCX 22 kb) [file 12916_2019_1353_MOESM4_ESM.docx]

**Additional File 4: Summary of Limitations of PHMRC Data**

A Critique of the PHMRC data used for assessments of automated VA COD classification methods

- Quality-related issues with the data:
  - Data inconsistencies/contradictories:
    - About 66% of deaths had respiratory symptoms identified, yet only 18% of the deaths were respiratory-related (Byass, 2014)
    - 147 stillborn deaths described as neonatal deaths in VA interviews (Byass, 2014)
    - 20% of maternal deaths not described as being pregnant or recently delivered (Byass, 2014)
    - All hierarchical algorithms assessed in Kalter et al. (2016) performed poorly when classifying ‘Measles’ for neonatal and child deaths due to lack of responses for rash, which was only reported in 3 of the 23 reference standard measles cases
  - Serina et al. (2016) found question reliability to be low and high response incompleteness between the initial and repeat interviews; though concluding that it does not affect Tariff 2.0 (moderate 0.61 kappa), this could potentially affect the performance of other data-driven COD classification algorithms that require training in order to classify records
  - Cause list categories and definitions:
    - Categories are arbitrary (grouping of many causes in ‘Other cause’) (Garenne, 2014)
    - Cause definitions differ from WHO ICD-10 definitions (Byass, 2014)
    - Not all algorithms produce an ill-defined or indeterminate category which captures the deceased records that may not be complete or accurate enough, or too complex, or else, have a pathology that could not be identified via interviews for COD classification
  - Different epidemiologic profiles captured:
    - Potentially excludes certain causes (causes that are unlikely to result in death due to receiving treatment) that are common in community settings (Garenne, 2014)
    - Different symptomatology and pathogen distributions captured, which may not be generalizable for rural or medically unattended deaths (Miasnikof et al., 2015; Byass, 2014; Garenne, 2014)
- Use has led to some implausible results:
  - Studies that report on the comparison of performance between algorithms and physician COD classification (PCVA) are unclear on the number of physicians involved for COD classification
    - Often assumed that single physician COD assignment was used as opposed to the standard practice of using dual physician review
    - Examples of papers lacking clarity or mentioning single physician COD assignment include: Murray et al., 2014; Flaxman et al., 2011; James et al., 2011; Lozano et al., 2011
  - Garenne (2014) had the following criticisms with regards to the Murray et al. (2014) findings:
    - The values for certain physician diagnoses were abnormally low
      - Sensitivity was only 41% for congenital defects, 39% for measles, 45% for accidental falls and 62% for maternal deaths
    - The sensitivity and positive predictive values (PPVs) for the algorithms had abnormally low values for specific diseases that are ‘easy to diagnose’
      - For example, algorithms and physician sensitivity values for the experiments without healthcare experience variables were less than 60% for prematurity, less than 40% for child diarrhoea and less than 50% for adult epilepsy or asthma
- Problems of external validity:
  - Limited in time and geographic scope
    - Deaths occurred in hospital: population covered are those who access health services, and so, responses will be different from respondents who did not access services/where deaths were unattended (Kalter et al., 2016; McCormick et al., 2016; Murray et al., 2014)
    - Not suitable for training and testing on more current community VAIs (post-2012) because prevalence rates of diseases change considerably over time and area (McCormick et al., 2016)
      - PHMRC data was collected from 2007 to 2010 within certain sites: Andhra Pradesh and Uttar Pradesh in India, Distrito Federal in Mexico, Bohol in the Philippines, and Dar es Salaam and Pemba (North and South) in Tanzania

**References**

Aleksandrowicz L, Malhotra V, Dikshit R, Gupta PC, Kumar R, Sheth J. Performance criteria for verbal autopsy-based systems to estimate national causes of death: development and application to the Indian Million Death Study. *BMC Medicine* 2014; **12**(21).

Byass P, Chandramohan D, Clark SJ, D'Ambruoso L, Fottrell E, Graham WJ, et al. Strengthening standardised interpretation of verbal autopsy data: the new InterVA-4 tool. *Glob Health Action* 2012; **5**. DOI: 10.3402/gha.v5i0.19281.

Byass P. Usefulness of the Population Health Metrics Research Consortium gold standard verbal autopsy data for general verbal autopsy methods. BMC medicine 2014; **12**(1): 23.

Garenne M. Prospects for automated diagnosis of verbal autopsies. BMC medicine 2014; **12**(1): 18.

Gomes M, Begum R, Sati P, et al. Nationwide Mortality Studies to Quantify Causes of Death: Relevant Lessons from India’s Million Death Study. *Health Aff (Millwood)* 2017; **36**: 1887-95.

Flaxman AD, Vahdatpour A, James SL, Birnbaum JK, Murray CJL. Direct estimation of cause-specific mortality fractions from verbal autopsies: multisite validation study using clinical diagnostic gold standards. *Population Health Metrics* 2011; **9**(35).

Fleiss JL, Cohen J. The equivalence of weighted kappa and the intraclass correlation coefficient as measures of reliability. *Educational and Psychological Measurement* 1973; **33**(3): 613-619. DOI: http://dx.doi.org/10.1177/001316447303300309

Institute for Health Metrics and Evaluation [IHME]. Verbal Autopsy Tools. http://www.healthdata.org/verbal-autopsy/tools [Accessed 31 Jan 2018a].

IHME. Population Health Metrics Research Consortium Gold Standard Verbal Autopsy Data 2005-2011. <http://ghdx.healthdata.org/record/population-health-metrics-research-consortium-gold-standard-verbal-autopsy-data-2005-2011> [Accessed 22 May 2018b].

IHME. (n.d.). SmartVA-Analyze 1.2 Help. <http://www.healthdata.org/sites/default/files/files/Tools/SmartVA/2017/Smartva-Analyze-v1.2_Help.pdf> [Accessed 2 Feb 2018].

Joint United Nations Programme on HIV/AIDS. Prevention GAP Report, 2016. Available from: http://www.unaids.org/en/resources/documents/2016/prevention-gap [Accessed 16 May 2017].

James SL, Flaxman AD, Murray CJL. Performance of the Tariff Method: validation of a simple additive algorithm for analysis of verbal autopsies. *Population Health Metrics* 2011; **9**(31).

Kalter HD, Perin J, Black RE. Validating hierarchical verbal autopsy expert algorithms in a large data set with known causes of death. *Journal of Global Health* 2016; **6**(1): 010601.

King G, Lu Y. Verbal autopsy methods with multiple causes of death. *Statistical Science* 2008; **23**(1): 78-91.

Leitao J, Desai N, Aleksandrowicz L, Byass P et al. Comparison of physician-certified verbal autopsy with computer-coded verbal autopsy for cause of death assignment in hospitalized patients in low-and middle-income countries: systematic review. *BMC Med* 2014; **12**(22).

Li Z. openVA: Automated Method for Verbal Autopsy. 2017. Available from: https://cran.r-project.org/package=openVA [Accessed 19 September 2017].

Li Z. InSilicoVA: Probabilistic Verbal Autopsy Coding with 'InSilicoVA' Algorithm. 2017. Available from: https://cran.r-project.org/package= InSilicoVA [Accessed 19 September 2017].

Li Z. InterVA4: Replicate and Analyse 'InterVA4'. 2017. Available from: https://cran.r-project.org/package=InterVA4 [Accessed 18 September 2017].

Lozano R, Lopez AD, Atkinson C, Naghavi M, Flaxman AD, Murray CJL. Performance of physician-certified verbal autopsies: multisite validation study using clinical diagnostic gold standards. *Population Health Metrics* 2011; **9**(32).

McCormick TH, Li ZR, Calvert C, Crampin AC, Kahn K, Clark SJ. Probabilistic Cause-of-Death Assignment Using Verbal Autopsies. *Journal of the American Statistical Association* 2016; **111**(515): 1036-1049; DOI: <https://doi.org/10.1080/01621459.2016.1152191>

Miasnikof P, Giannakeas V, Gomes M, et al. Naive Bayes classifiers for verbal autopsies: comparison to physician-based classification for 21,000 child and adult deaths. *BMC Medicine* 2015; **13**(1): 286.

Murray CJ, Lozano R, Flaxman AD, et al. Using verbal autopsy to measure causes of death: the comparative performance of existing methods. *BMC Med* 2014; **12**(5).

Serina P, Riley I, Stewart A, James SL, et al. Improving performance of the Tariff Method for assigning causes of death to verbal autopsies. *BMC Medicine* 2015; **13**(291); doi: <https://doi.org/10.1186/s12916-015-0527-9>

Sinha DN, Dikshit R, Kumar V, Gajalakshmi V, Dhingra N, Seth J. Technical document VII: Health care professional's manual for assigning causes of death based on RHIME household reports. Toronto: Centre for Global Health Research, University of Toronto, 2006.

Wen R. nbc4va: Bayes Classifier for Verbal Autopsy Data. 2016. Available from: https://cran.r-project.org/package=nbc4va [Accessed 18 September 2017].

World Health Organization. International statistical classifications of diseases and related health problems, 10th rev. Volume 1 Geneva, Switzerland: World Health Organization, 2008.

World Health Organization. World Malaria Report 2016. Geneva: World Health Organization; 2016. Available from: http://www.who.int/malaria/publications/world-malaria-report-2016/report [Accessed 16 May 2017].
