# Supplementary material for: Automated versus physician assignment of cause of death for verbal autopsies: randomized trial of 9374 deaths in 117 villages in India
Source: BMC Med. 2019 Jun 27;17:116. doi: 10.1186/s12916-019-1353-2 (PMC6595581; doi:10.1186/s12916-019-1353-2)
Supplement: Supplementary file 5 — Cause of death categories with corresponding ICD-10 codes. (DOCX 23 kb) [file 12916_2019_1353_MOESM5_ESM.docx]

**Additional File 5: Cause of death categories with corresponding ICD-10 codes**

| Cause of death | ICD-10 Range |
| --- | --- |
| Adult (12-69 years) |  |
| Acute respiratory infections | H65-H68, H70-H71, J00-J22, J32, J36, J85-J86, P23 |
| Tuberculosis | A15-A16, B90, J65 |
| Diarrhoeal diseases | A00-A09 |
| Unspecified infections | A17-A33, A35-A99, B00-B17, B19-B89, B91-B99, C46, D64, D84, G00-G09, H10, H60, I30, I32-I33, K02, K04-K05, K61, K65, K67, K81, L00-L04, L08, M00-M01, M60, M86, N10, N30, N34, N41, N49, N61, N70-N74, P35-P39, R50, R75, ZZ21 |
| Maternal conditions | A34, F53, O00-O08, O10-O16, O20-O99 |
| Nutritional deficiencies | D50-D53, E00-E02, E40-E46, E50-E64, X53-X54 |
| Chronic respiratory diseases | J30-J31, J33-J35, J37-J64, J66-J84, J90-J99, R04-R06, R84, R91 |
| Cancers | C00-C26, C30-C45, C47-C58, C60-C97, D00-D48, D91, N60, N62-N64, N87, R59 |
| Ischemic heart disease | I20-I25, R55 |
| Stroke | G45-G46, G81-G83, I60-I69 |
| Diabetes mellitus | E10-E14 |
| Other cardiovascular diseases | I00-I03, I05-I15, I26-28, I31, I34-I52, I70-I99, R00-R01, R03, ZZ23 |
| Liver and alcohol related diseases | B18, F10, K70-K77, R16-R18, X45, Y15, Y90-91 |
| Other noncommunicable diseases | D55-D63, D65-D83, D86, D89, E03-E07, E15-E35, E65-E68, E70-E90, F00-F09, F11-F52, F54-F99, G10-G37, G40-G41, G50-G80, G84-G99, H00-H06, H11-H59, H61-H62, H69, H72-H95, K00-K01, K03, K06-K14, K20-K31, K35-K38, K40-K60, K62-K64, K66, K78-K80, K82-K93, L05, L10-L99, M02-M54, M61-M85, M87-M99, N00-N08, N11-N29, N31-N33, N35-N40, N42-N48, N50-N59, N75-N86, N88-N99, Q00-Q99, R10-R15, R19-R23, R26-R27, R29-R49, R56, R63, R70-R74, R76-R77, R80-R82, R85-R87, R90, ZZ25 |
| Road and transport injuries | V01-V99, Y85 |
| Suicide | X60-X84 |
| Other injuries | S00-S99, T00-T99, W00-W99, X00-X44, X46-X52, X55-X59, X85-X99, Y00-Y14, Y16-Y84, Y86-Y89, Y92-Y98, ZZ27 |
| Ill-defined | R02, R07-R09, R25, R51-R54, R57-R58, R60-R62, R64-R69, R78-R79, R83, R89, R92-R94, R96, R98-R99 |
| Child (28 days - 11 years) |  |
| Pneumonia | A37, H65-H68, H70-H71, J00-J22, J32, J36, J85-J86, P23, U04 |
| Diarrhoeal diseases | A00-A09 |
| Malaria | B50-B54 |
| Other infections | A15-A28, A30-A36, A38-A44, A46, A48-A71, A74-A75, A77-A99, B00-B09, B15-B27, B30, B33-B49, B55-B60, B64-B83, B85-B92, B94-B97, B99, G00-G09, H10, H60, I30, I32-I33, I39-I41, J65, K02, K04-K05, K61, K65, K67, K81, L00-L04, L08, M00-M01, M60, M86, N10, N30, N34, N41, N49, N61, N70-N74, P35-P39, R50, R75, U00, Y95, ZZ11 |
| Congenital anomalies | P01, P05, P07, P21, Q00-Q99 |
| Epilepsy, leukaemia, and other noncommunicable diseases | C00-C97, D01-D48, D55-D89, E03-E35, E65-E90, F00-F02, F73, G10-G99, H00-H06, H11-H59, H61-H62, H69, H72-H95, I00-I28, I31, I34-I38, I42-I99, J30-J31, J33-J35, J37-J47, J60, J64, J66-J70, J80-J82, J84, J90-J99, K00-K01, K03, K06-K60, K62-K63, K70-K80, K82-K93, L05, L10-L99, M02-M54, M61-M85, M87-M99, N00-N08, N11-N29, N31-N33, N35-N40, N42-N48, N50-N51, N60, N62-N64, N75-N99, P04, P08, P27, P51, P53-P60, P70-P72, P74-P76, P78, P80-P83, P92-P94, R00-R01, R03-R06, R11-R23, R26-R27, R29-R49, R55-R56, R59, R63, R70-R74, R76-R77, R80-R82, R84-R87, R90-R91, ZZ12-ZZ13, ZZ15 |
| Injuries | S00-S99, T00-T98, V01-V99, W00-W99, X00-X52, X57-X99, Y00-Y91, Y97-Y98 |
| Nutritional deficiencies | D50-D53, E00-E02, E40-E46, E50-E56, E59-E61, E63-E64, X53-X54 |
| Other | D00, F03-F72, F74-F99, P00, P02-P03, P10-P15, P20, P22, P24-P26, P28-P29, P50, P52, P61, P77, P90-P91 |
| Ill-defined | P96, R02, R07, R09-R10, R25, R51-R54, R57-R58, R60-R62, R64, R68-R69, R78-R79, R83, R89, R92-R99 |
| Neonate (0-27 days) |  |
| Prematurity and low birthweight | D64, O60, P01, P05, P07, P22, P25-P28, P52, P61, P77, P80, P92, R04 |
| Neonatal infections | A00-A09, A20-A28, A32-A35, A37-A44, A46, A48-A49, A68-A70, A74-A75, A77-A79, A81-A90, B54, B95-B96, G00-G09, H10, H60, H65-H68, H70-H71, I30, I32-I33, I39-I41, J00-J22, J32, J36, J85-J86, K65, K67, K81, L00-L04, L08, M00-M01, M60, M86, N10, N30, N34, N41, N49, N61, O85, P23, P35-P39, P58-P59, U04 |
| Birth asphyxia and birth trauma | G40, P00, P02-P03, P10-P15, P20-P21, P24, P29, P50-P51, P90-P91, R06, W79, Z37 |
| Congenital anomalies | C76, Q00-Q99 |
| Other | A15-A19, A30-A31, A36, A50-A67, A71, A80, A91-A99, B00-B09, B15-B27, B30, B33-B53, B55-B60, B64-B83, B85-B92, B94, B97, B99, C00-C75, C77-C97, D00-D48, D50-D53, D55-D63, D65-D89, E00-E35, E40-E46, E50-E56, E59-E61, E63-E90, F00-F99, G10-G39, G41-G99, H00-H06, H11-H59, H61-H62, H69, H72-H95, I00-I28, I31, I34-I38, I42-I99, J30, J31, J33-J35, J37-J47, J60, J64-J70, J80-J82, J84, J90-J99, K00-K63, K70-K80, K82-K93, L05, L10-L99, M02-M54, M61-M85, M87-M99, N00-N08, N11-N29, N31-N33, N35-N40, N42-N48, N50-N51, N60, N62-N64, N70-N99, P04, P08, P53-P57, P60,P70-P72, P74-P76, P78, P81-P83, P93-P94, R00-R01, R03-R05, R11-R23, R26-R27, R29-R36, R39-R50, R55-R56, R59, R63, R70-R77, R80-R82, R84-R87, R90-R91, S00-S99, T00-T98, U00, V01-V99, W00-W78, W80-W99, X00-X54, X57-X99, Y00-Y91, Y95, Y97-Y98 |
| Ill-defined | P96, R02, R07, R09-R10, R25, R51-R54, R57-R58, R60-R62, R64, R68-R69, R78-R79, R83, R89, R92-R99 |
