# Supplementary material for: Automated versus physician assignment of cause of death for verbal autopsies: randomized trial of 9374 deaths in 117 villages in India
Source: BMC Med. 2019 Jun 27;17:116. doi: 10.1186/s12916-019-1353-2 (PMC6595581; doi:10.1186/s12916-019-1353-2)
Supplement: Supplementary file 6 — Geographic and age distribution of deaths below age 70 years used in analyses by study group. (DOCX 20 kb) [file 12916_2019_1353_MOESM6_ESM.docx]

**Additional File 6: Geographic and age distribution of deaths below age 70 years used in analyses by study group**

|  | ***Pilot Study Site*** | | ***Main Randomized Trial Sites*** | | | | | |
| --- | --- | --- | --- | --- | --- | --- | --- | --- |
|  | **Maharashtra** | | **Gujarat** | | **Punjab** | | **TOTAL** | |
|  | **Physician assignment** | **Automated assignment** | **Physician assignment** | **Automated assignment** | **Physician assignment** | **Automated assignment** | **Physician assignment** | **Automated assignment** |
| **Adult** | 527 | 578 | 2410 | 2457 | 1901 | 1936 | **4311** | **4393** |
| **Child** | 35 | 31 | 107 | 104 | 83 | 109 | **190** | **213** |
| **Neonate** | 27 | 17 | 45 | 51 | 105 | 66 | **150** | **117** |
| **TOTAL** | **589** | **626** | **2562** | **2612** | **2089** | **2111** | **4651** | **4723** |

The trial was conducted in a total of 5 districts and 3 states of India. The first state, Maharashtra, was the pilot state (Amravati district), meanwhile, the other 4 trials occurred in Anand and Kheda districts in Gujarat, and Sangrur and Mansa districts in Punjab.
