# Supplementary material for: Automated versus physician assignment of cause of death for verbal autopsies: randomized trial of 9374 deaths in 117 villages in India
Source: BMC Med. 2019 Jun 27;17:116. doi: 10.1186/s12916-019-1353-2 (PMC6595581; doi:10.1186/s12916-019-1353-2)
Supplement: Supplementary file 7 — Summary of cause list matches between all algorithm results and the cause of death categories used in this study. (DOCX 19 kb) [file 12916_2019_1353_MOESM7_ESM.docx]

**Additional File 7: Summary of cause list matches between all algorithm results and the cause of death categories used in this study**

|  | NBC | King-Lu | SmartVA | InSilicoVA | InSilicoVA-NT | InterVA-4 |
| --- | --- | --- | --- | --- | --- | --- |
| Adults (N = 18) | | | | | | |
| Cause list | 16 | 16 | 34 | 18 | 48 | 43 |
| Final cause list | 16 | 16 | 17 | 16 | 17 | 18 |
| Children (N = 10) | | | | | | |
| Cause list | 7 | 7 | 18 | 10 | 32 | 28 |
| Final cause list | 7 | 7 | 7 | 7 | 8 | 8 |
| Neonates (N = 6) | | | | | | |
| Cause list | 4 | 4 | 4 | 6 | 7 | 8 |
| Final cause list | 4 | 4 | 4 | 4 | 5 | 5 |

Cause list denotes the total number of unique causes generated by each algorithm, whereas the final cause list refers to the total number of causes after mapping with the cause list categories outlined in web appendix Table 1 (N = total number of cause categories per age group).
