# Supplementary material for: Automated versus physician assignment of cause of death for verbal autopsies: randomized trial of 9374 deaths in 117 villages in India
Source: BMC Med. 2019 Jun 27;17:116. doi: 10.1186/s12916-019-1353-2 (PMC6595581; doi:10.1186/s12916-019-1353-2)
Supplement: Supplementary file 8 — Percent population-level concordance in cause of death distribution between algorithms of the automated assignment deaths, by age groups. (DOCX 20 kb) [file 12916_2019_1353_MOESM8_ESM.docx]

**Additional File 8: Percent population level concordance in cause of death distribution between algorithms of the automated assignment deaths, by age groups**

|  |  | *Require training data* | | | |  | *Do not require training data* | |
| --- | --- | --- | --- | --- | --- | --- | --- | --- |
| Age Group | Mean (SD) | NBC | King-Lu | SmartVA | InSilicoVA |  | InSilicoVA-NT | InterVA-4 |
| **Adult** | 62 (15) | 46 | 43 | 65 | 64 |  | 69 | 83 |
| **Child** | 58 (8) | 53 | 64 | 44 | 62 |  | 58 | 65 |
| **Neonate** | 63 (23) | 58 | 72 | 22 | 89 |  | 64 | 72 |

Mean and standard deviation (SD) of the population level concordance attained for the automated assignment VA algorithms when using data from all PHMRC sites as the training data, stratifying by age groups: adult (12-69 years); child (28 days – 11 years); and neonate (0-27 days). The algorithms were run on automated assignment trial data and the results were compared against the dual physician review of the automated assignment trial data. InSilicoVA-NT and InterVA-4 do not require training data, whereas SmartVA was pre-trained using data from all PHMRC sites.
