# Supplementary material for: Automated versus physician assignment of cause of death for verbal autopsies: randomized trial of 9374 deaths in 117 villages in India
Source: BMC Med. 2019 Jun 27;17:116. doi: 10.1186/s12916-019-1353-2 (PMC6595581; doi:10.1186/s12916-019-1353-2)
Supplement: Supplementary file 9 — Mean population-level concordance (%) comparison of verbal autopsy methods using non-randomized study data, by algorithm. (DOCX 111 kb) [file 12916_2019_1353_MOESM9_ESM.docx]

**Additional File 9: Mean population level concordance (%) comparison of verbal autopsy methods using non-randomized study data, by algorithm**


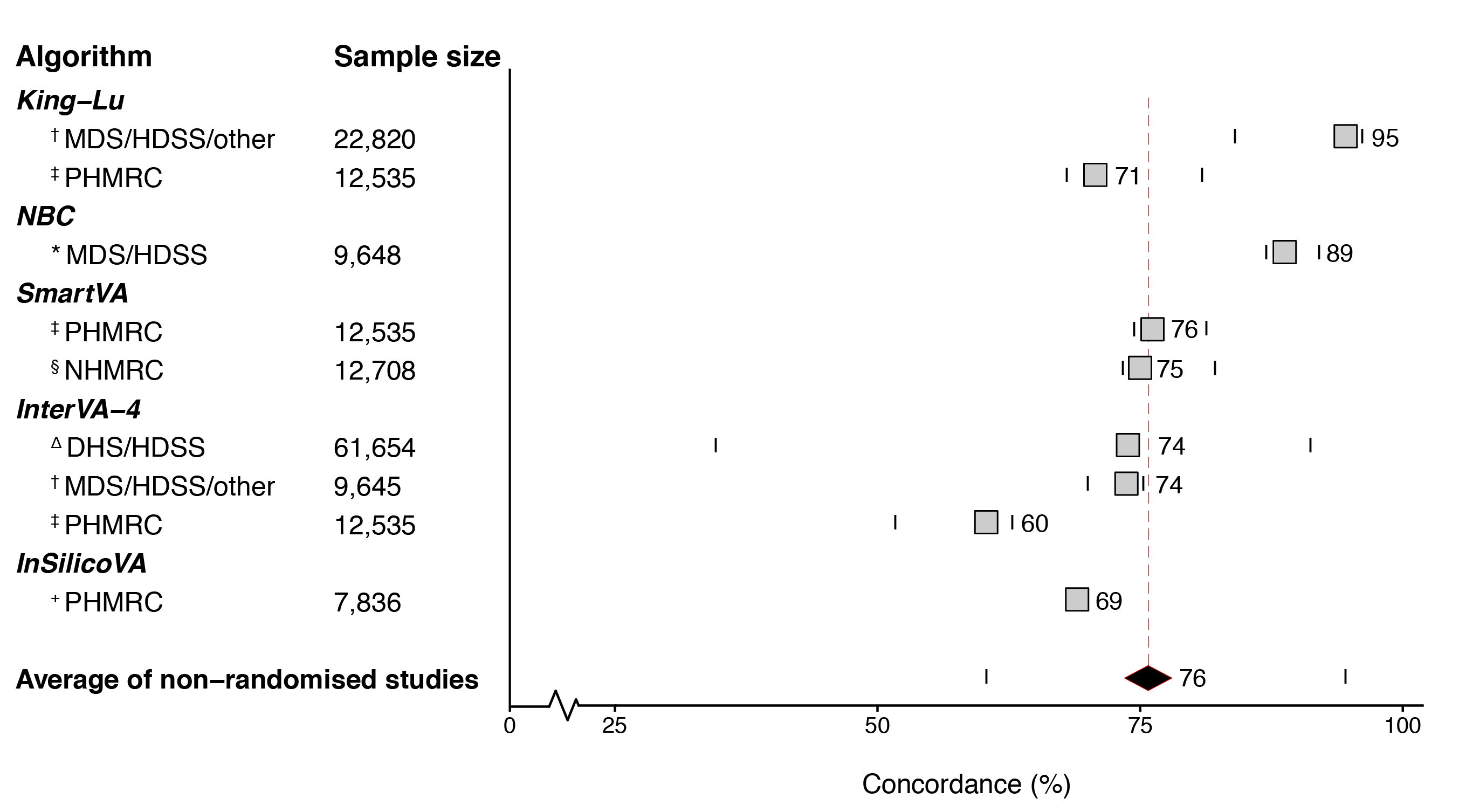


Mean concordance was weighted (sample size) and averaged (horizontal bars indicate the range of the weighted mean concordance estimates) from the following studies:

* Miasnikof et al. Naive Bayes classifiers for verbal autopsies: comparison to physician-based classification for 21,000 child and adult deaths. *BMC Medicine* 2015, 13(286)

^†^ Desai et al. Performance of four computer-coded verbal autopsy methods for cause of death assignment compared with physician coding on 24,000 deaths in low- and middle-income countries. *BMC Medicine* 2014, 12(20)

^‡^ Murray et al. Using verbal autopsy to measure causes of death: the comparative performance of existing methods. *BMC Medicine* 2014, 12(5)

^§^ Serina et al. Improving performance of the Tariff Method for assigning causes of death to verbal autopsies. *BMC Medicine* 2015, 13(291)

^+^ McCormick et al. Probabilistic Cause-of-death Assignment using Verbal Autopsies*. J AM Stat Assoc* 2016, 111(515): 1036-1049. Note: single experiment study with no range available.

Mean Concordance Correlation Coefficient (CCC) (with ranges) was presented due to absence of CSMF accuracies from the following studies:

^Δ^ Byass et al. Comparing verbal autopsy cause of death findings as determined by physician coding and probabilistic modeling: a public health analysis of 54,000 deaths in Africa and Asia. *Journal of Global Health* 2015, 5(1); Oti SO & Kyobutungi C. Verbal autopsy interpretation: a comparative analysis of the InterVA model versus physician review in determining causes of death in the Nairobi DSS. *Population Health Metrics* 2010, 8(21); Ramroth et al. Cause of death distribution with InterVA and physician coding in a rural area of Burkina Faso. *Tropical Medicine and International Health* 2012, 17(7):904-913.
