# Supplementary material for: Automated versus physician assignment of cause of death for verbal autopsies: randomized trial of 9374 deaths in 117 villages in India
Source: BMC Med. 2019 Jun 27;17:116. doi: 10.1186/s12916-019-1353-2 (PMC6595581; doi:10.1186/s12916-019-1353-2)
Supplement: Supplementary file 10 — Percent proportion of causes of death by age groups and training dataset: comparing physician assignment deaths versus the closest automated assignment proportion of deaths for the same cause. (DOCX 25 kb) [file 12916_2019_1353_MOESM10_ESM.docx]

**Additional File 10: Percent proportion of causes of death by age groups and training dataset: comparing physician assignment deaths versus the closest automated assignment proportion of deaths for the same cause**

| Cause of death | Standard (Physician assignment) | Automated assignment  *Global training data* | Automated assignment  *Indian training data* | Single Physician assigned  *Global training data (PHMRC^†^)* |
| --- | --- | --- | --- | --- |
| Adult (12-69 years) | **N=4311** | **N=4393** | **N=4393** | **N=4654** |
| Ischemic heart disease | 17.1 | 13.0 (InSilicoVA) | 12.8 (InSilicoVA-NT) | 3.7 |
| *Cancers** | 13.7 | 12.9 (InterVA-4) | 12.9 (InterVA-4) | 13.6 |
| Other noncommunicable diseases | 8.7 | 7.5 (InterVA-4) | 7.5 (InterVA-4) | 13.0 |
| Unspecified infections | 8.4 | 8.5 (InSilicoVA) | 9.0 (InterVA-4) | 13.4 |
| *Falls, bites & other injuries** | 7.6 | 9.3 (SmartVA) | 9.3 (SmartVA) | 10.1 |
| Tuberculosis | 7.0 | 10.1 (InSilicoVA-NT) | 6.0 (InSilicoVA) | 2.8 |
| Chronic respiratory diseases | 6.9 | 6.2 (InterVA-4) | 6.2 (InterVA-4) | 1.8 |
| *Road and transport injuries** | 6.4 | 6.4 (InterVA-4) | 6.4 (InterVA-4) | 2.7 |
| Stroke | 5.4 | 5.5 (InterVA-4) | 5.5 (InterVA-4) | 6.3 |
| *Suicide** | 4.8 | 5.5 (InSilicoVA) | 4.9 (NBC) | 1.5 |
| Liver and alcohol related diseases | 3.2 | 2.8 (InSilicoVA) | 1.7 (InSilicoVA-NT) | 5.0 |
| Other cardiovascular diseases | 2.5 | 3.1 (NBC) | 2.1 (NBC) | 5.2 |
| Acute respiratory infections | 2.3 | 1.1 (King-Lu) | 0.5 (SmartVA) | 6.5 |
| Diarrhoeal diseases | 2.2 | 2.8 (NBC) | 1.4 (SmartVA) | 2.2 |
| Diabetes mellitus | 1.5 | 1.3 (NBC) | 1.1 (InterVA-4) | 4.8 |
| Maternal conditions | 0.6 | 0.6 (InSilicoVA-NT) | 0.5 (NBC) | 7.4 |
| Nutritional deficiencies | 0.1 | 0.0 (2 or more) | 0.0 (2 or more) | 0.0 |
| Ill-defined | 1.6 | 0.0 (2 or more) | 0.0 (2 or more) | 0.0 |
| *Agreement* |  | ***98.3*** | ***91.4*** |  |
| Child (28 days - 11 years) | **N=190** | **N=213** | **N=213** | **N= 2064** |
| Epilepsy, leukaemia & other noncommunicable diseases | 22.6 | 23.9 (King-Lu) | 22.1 (InSilicoVA-NT) | 14.1 |
| Other infections | 16.8 | 10.3 (InSilicoVA) | 15.5 (InSilicoVA) | 12.6 |
| *Road & transport injuries, drowning & other injuries** | 14.2 | 13.1 (SmartVA) | 13.1 (SmartVA) | 20.2 |
| Pneumonia | 13.7 | 17.8 (NBC) | 15.7 (InSilicoVA) | 25.8 |
| Diarrhoeal diseases | 11.1 | 11.7 (SmartVA) | 11.7 (SmartVA) | 12.4 |
| Congenital anomalies | 10.0 | 3.5 (InSilicoVA-NT) | 3.4 (InSilicoVA-NT) | 0.0 |
| Malaria | 5.3 | 4.7 (InterVA-4) | 4.7 (InterVA-4) | 5.6 |
| Nutritional deficiencies | 2.1 | 1.1 (InSilicoVA-NT) | 1.1 (InSilicoVA-NT) | 0.0 |
| Other | 0.0 | 0.0 (2 or more) | 0.0 (2 or more) | 9.4 |
| Ill-defined | 4.2 | 0.0 (InSilicoVA-NT) | 0.0 (InSilicoVA-NT) | 0.0 |
| *Agreement* |  | ***93.0*** | ***94.0*** |  |
| Neonate (0-27 days) | **N=150** | **N=117** | **N=117** | **N=1504** |
| Prematurity and low birthweight | 30.0 | 33.3 (InterVA-4) | 31.9 (InSilicoVA) | 41.2 |
| Birth asphyxia and birth trauma | 28.7 | 29.5 (King-Lu) | 38.6 (King-Lu) | 27.6 |
| Congenital anomalies | 16.7 | 13.7 (SmartVA) | 14.3 (King-Lu) | 15.0 |
| Neonatal infections | 10.7 | 16.8 (InSilicoVA) | 7.1 (InSilicoVA) | 16.3 |
| Other | 0.0 | 0.0 (2 or more) | 0.0 (2 or more) | 0.0 |
| Ill-defined | 14.0 | 0.0 (2 or more) | 0.0 (2 or more) | 0.0 |
| *Agreement* |  | ***96.2*** | ***95.4*** |  |

The percentage of causes of death for the dual physician COD assignment deaths were compared against the percentages generated by the automated COD assignment VA algorithms, and the algorithm with smallest absolute difference per cause of death per age group is recorded in the table above. Population level concordance was computed using the cause-specific percentages with the smallest absolute difference regardless of algorithm per age group. The algorithms were run using data from all PHMRC sites versus only the Indian sites as training data. InSilicoVA-NT, InterVA-4, and SmartVA do not require training data; hence, the cause of death proportions generated by these algorithms are unchanged when the training dataset is varied. * More obvious diagnoses. The order of injuries for adults, from highest to lowest number of deaths, is as follows: falls, other injuries, bites. † The percentage of causes of death for the single physician assignment deaths per age group were calculated from the Population Health Metrics Research Consortium (PHMRC) Institute for Health Metrics and Evaluation (IHME) data from which the training data were derived. Global training data included data from all sites compared to only Indian sites (Andhra Pradesh and Uttar Pradesh) for the Indian training data. The concordance between PHMRC global data and standard physician assignment data per age group is 68%, 75%, and 81% for adult, child, and neonate age groups, respectively.
