# Supplementary material for: Automated versus physician assignment of cause of death for verbal autopsies: randomized trial of 9374 deaths in 117 villages in India
Source: BMC Med. 2019 Jun 27;17:116. doi: 10.1186/s12916-019-1353-2 (PMC6595581; doi:10.1186/s12916-019-1353-2)
Supplement: Supplementary file 11 — Cause of death counts, proportions, and rankings for adults with dual physician coding of the automated arm. (DOCX 27 kb) [file 12916_2019_1353_MOESM11_ESM.docx]

**Additional File 11: Cause of death counts, proportions and rankings for adults with dual physician coding of the automated arm**

| Rank | Cause of death | No. of deaths | | | | | |  | Standard (physician assigned), % | | Proportion, % (rank) | | | | | | | |
| --- | --- | --- | --- | --- | --- | --- | --- | --- | --- | --- | --- | --- | --- | --- | --- | --- | --- | --- |
|  |  | Standard (physician-assigned) | | Dual physician review estimated deaths | Mean algorithm estimated deaths | | 2 or more algorithms agreed |  |  |  |  | *Require training data* | | |  | *Do not require training data* | | Dual Physician review of automated assignment arm |
|  |  | Total | Both physicians initially agreed† |  |  |  |  |  |  |  | NBC | King-Lu | SmartVA | InSilicoVA |  | InSilicoVA-NT | InterVA-4 |  |
|  |  |  |  |  |  |  |  |  |  |  |  |  |  |  |  |  |  |  |
|  | **Adult (12-69 years)** | |  |  | |  |  |  | |  |  |  |  |  |  |  |  |  |
| 1 | Ischemic heart disease | 737 | 661 | 522 | 368 | | 506 |  | 17.1 | | 5.1 (5) | 8.3 (5) | 4.0 (8) | 13.0 (2) |  | 12.8 (2) | 8.0 (5) | 12.1 (2) |
| 2 | *Cancers** | 592 | 517 | 543 | 287 | | 393 |  | 13.7 | | 2.0 (12) | 1.6 (10) | 8.4 (3) | 4.4 (10) |  | 10.6 (4) | 12.9 (1) | 12.6 (1) |
| 3 | Other noncommunicable diseases | 376 | 273 | 285 | 314 | | 365 |  | 8.7 | | 6.1 (4) | 0.2 (16) | 6.1 (5) | 6.6 (6) |  | 17.2 (1) | 7.5 (6) | 6.6 (7) |
| 4 | Unspecified infections | 363 | 265 | 366 | 242 | | 278 |  | 8.4 | | 1.8 (13) | 0.7 (15) | 1.3 (14) | 8.5 (4) |  | 12.4 (3) | 9.0 (4) | 8.5 (5) |
| 5 | *Falls, bites, and other injuries** | 328 | 288 | 302 | 811 | | 401 |  | 7.6 | | 50.4 (1) | 32.7 (1) | 9.3 (2) | 4.6 (9) |  | 10.1 (6) | 5.8 (9) | 7.0 (6) |
| 6 | Tuberculosis | 303 | 252 | 216 | 211 | | 283 |  | 7 | | 3.9 (7) | 1.9 (8) | 2.3 (10) | 0.2 (16) |  | 10.1 (5) | 10.9 (3) | 5.0 (10) |
| 7 | Chronic respiratory diseases | 296 | 231 | 479 | 175 | | 235 |  | 6.9 | | 1.0 (15) | 0.9 (14) | 8.1 (4) | 3.8 (11) |  | 4.3 (9) | 6.2 (8) | 11.1 (3) |
| 8 | *Road and transport injuries** | 274 | 246 | 246 | 491 | | 288 |  | 6.4 | | 6.6 (2) | 14.9 (2) | 5.6 (6) | 27.8 (1) |  | 7.0 (7) | 6.4 (7) | 5.7 (8) |
| 9 | Stroke | 232 | 196 | 224 | 160 | | 197 |  | 5.4 | | 2.2 (11) | 3.9 (7) | 5.2 (7) | 4.8 (8) |  | 0.7 (15) | 5.5 (10) | 5.2 (9) |
| 10 | *Suicide** | 208 | 185 | 129 | 200 | | 109 |  | 4.8 | | 3.9 (6) | 11.7 (3) | 1.8 (11) | 5.5 (7) |  | 1.9 (11) | 3.1 (13) | 3.0 (13) |
| 11 | Liver and alcohol related diseases | 137 | 105 | 147 | 63 | | 67 |  | 3.2 | | 0.9 (16) | 1.5 (11) | 0.5 (16) | 2.8 (12) |  | 1.6 (12) | 1.4 (14) | 3.4 (12) |
| 12 | Other cardiovascular diseases | 108 | 64 | 60 | 165 | | 166 |  | 2.5 | | 3.1 (8) | 1.2 (12) | 1.6 (12) | 8.5 (3) |  | 3.7 (10) | 4.8 (11) | 1.4 (15) |
| 13 | Acute respiratory infections | 100 | 72 | 99 | 127 | | 114 |  | 2.3 | | 6.1 (3) | 1.1 (13) | 0.5 (17) | 0.5 (14) |  | 4.8 (8) | 4.7 (12) | 2.3 (14) |
| 14 | Diarrhoeal diseases | 96 | 68 | 207 | 139 | | 64 |  | 2.2 | | 2.8 (10) | 6.3 (6) | 1.4 (13) | 7.9 (5) |  | 0.4 (17) | 0.5 (17) | 4.8 (11) |
| 15 | Ill-defined | 69 | 59 | 453 | 366 | | 194 |  | 1.6 | | 0.0 (18) | 0.0 (18) | 39.7 (1) | 0.0 (18) |  | 0.0 (18) | 11.3 (2) | 10.5 (4) |
| 16 | Diabetes mellitus | 63 | 49 | 22 | 64 | | 35 |  | 1.5 | | 1.3 (14) | 1.7 (9) | 3.6 (9) | 0.3 (15) |  | 0.9 (13) | 1.1 (15) | 0.5 (16) |
| 17 | Maternal conditions | 25 | 21 | 13 | 121 | | 29 |  | 0.6 | | 3.0 (9) | 11.4 (4) | 0.8 (15) | 0.6 (13) |  | 0.6 (16) | 0.4 (18) | 0.3 (17) |
| 18 | Nutritional deficiencies | 4 | 3 | 0 | 10 | | 4 |  | 0.1 | | 0.0 (17) | 0.0 (17) | 0.0 (18) | 0.0 (17) |  | 0.8 (14) | 0.6 (16) | 0.0 (18) |
|  | ***Agreement*** |  |  |  |  | |  |  |  | | ***50*** | ***44*** | ***57*** | ***66*** |  | ***77*** | ***80*** | ***84*** |

* More obvious diagnoses. The order of injuries in this category, from highest to lowest number of deaths, is: falls, other injuries, bites. † Percentage of agreement between both physicians at initial stage of ICD coding, where both physicians assigned the same cause of death for the deceased record. The overall physician initial agreement for adult was 83%.
