# Supplementary material for: Automated versus physician assignment of cause of death for verbal autopsies: randomized trial of 9374 deaths in 117 villages in India
Source: BMC Med. 2019 Jun 27;17:116. doi: 10.1186/s12916-019-1353-2 (PMC6595581; doi:10.1186/s12916-019-1353-2)
Supplement: Supplementary file 12 — Cause of death counts, proportions, and rankings for child and neonate age groups with dual physician coding of the automated arm. (DOCX 27 kb) [file 12916_2019_1353_MOESM12_ESM.docx]

**Additional File 12: Cause of death counts, proportions and rankings for child and neonate age groups with dual physician coding of the automated arm**

| Rank | Cause of death | No. of deaths | | | | | |  | | Standard (physician assigned), % | | Proportion, % (rank) | | | | | | | |
| --- | --- | --- | --- | --- | --- | --- | --- | --- | --- | --- | --- | --- | --- | --- | --- | --- | --- | --- | --- |
|  |  | Standard (physician-assigned) | | Dual physician review estimated deaths | Mean algorithm estimated deaths | | 2 or more algorithms agreed |  | |  |  |  | *Require training data* | | |  | *Do not require training data* | | Dual Physician review of automated assignment arm |
|  |  | Total | Both physicians initially agreed† |  |  |  |  |  | |  |  | NBC | King-Lu | SmartVA | InSilicoVA |  | InSilicoVA-NT | InterVA-4 |  |
|  |  |  |  |  |  |  |  |  | |  |  |  |  |  |  |  |  |  |  |
|  | **Child (28 days – 11 years)** | |  |  |  | |  | |  | |  |  |  |  |  |  |  |  |  |
| 1 | Epilepsy, leukaemia & other noncommunicable diseases | 43 | 33 | 38 | 26 | | 14 |  | | | 22.6 | 4.7 (5) | 23.9 (2) | 1.9 (7) | 9.8 (5) |  | 25.9 (2) | 16.4 (2) | 20.0 (1) |
| 2 | Other infections | 32 | 27 | 19 | 32 | | 27 |  | | | 16.8 | 1.9 (7) | 5.3 (6) | 3.8 (4) | 10.3 (4) |  | 44.1 (1) | 35.7 (1) | 10.0 (5) |
| 3 | *Road traffic accidents, drowning, and other injuries** | 27 | 22 | 31 | 49 | | 80 |  | | | 14.2 | 38.5 (1) | 41.2 (1) | 13.1 (2) | 27.9 (1) |  | 17.9 (3) | 15.5 (4) | 16.2 (2) |
| 4 | Pneumonia | 26 | 17 | 13 | 14 | | 10 |  | | | 13.7 | 17.8 (3) | 4.8 (7) | 2.3 (6) | 19.5 (2) |  | 0.0 (9) | 0.0 (9) | 6.7 (8) |
| 5 | Diarrhoea | 21 | 18 | 26 | 20 | | 33 |  | | | 11.1 | 24.9 (2) | 6.1 (5) | 11.7 (3) | 6.6 (7) |  | 6.5 (4) | 7.5 (5) | 13.8 (4) |
| 6 | Congenital anomalies | 19 | 15 | 27 | 1 | | 1 |  | | | 10 | 0.0 (8) | 0.0 (8) | 0.0 (10) | 0.0 (8) |  | 3.5 (5) | 0.9 (8) | 14.3 (3) |
| 7 | Malaria | 10 | 7 | 16 | 14 | | 20 |  | | | 5.3 | 8.5 (4) | 11.9 (3) | 0.0 (8) | 17.1 (3) |  | 1.0 (7) | 4.7 (6) | 8.6 (7) |
| 8 | Ill-defined | 8 | 6 | 18 | 25 | | 7 |  | | | 4.2 | 0.0 (10) | 0.0 (10) | 63.4 (1) | 0.0 (10) |  | 0.0 (8) | 16.0 (3) | 9.5 (6) |
| 9 | Nutritional | 4 | 2 | 2 | 1 | | 1 |  | | | 2.1 | 0.0 (9) | 0.0 (9) | 0.0 (9) | 0.0 (9) |  | 1.1 (6) | 3.3 (7) | 1.0 (9) |
| 10 | Other | 0 | 0 | 0 | 7 | | 6 |  | | | 0 | 3.8 (6) | 6.8 (4) | 3.8 (5) | 8.8 (6) |  | 0.0 (10) | 0.0 (10) | 0.0 (10) |
|  | ***Agreement*** |  |  |  |  | |  |  | | |  | ***51*** | ***58*** | ***36*** | ***60*** |  | ***66*** | ***66*** | ***82*** |
|  | ***Neonate (0 – 27 days)*** | | |  |  | |  | |  | |  |  |  |  |  |  |  |  |  |
| 1 | Prematurity & low birthweight | 45 | 31 | 47 | | 37 | 21 |  | | | 30 | 21.4 (2) | 15.6 (4) | 4.3 (3) | 34.4 (2) |  | 40.9 (1) | 33.3 (2) | 31.6 (1) |
| 2 | Birth asphyxia & birth trauma | 43 | 29 | 44 | | 41 | 62 |  | | | 28.7 | 66.7 (1) | 29.5 (2) | 2.6 (4) | 35.6 (1) |  | 13.1 (3) | 16.2 (3) | 29.1 (2) |
| 3 | Congenital anomalies | 25 | 20 | 19 | | 17 | 8 |  | | | 16.7 | 10.3 (3) | 21.6 (3) | 13.7 (2) | 13.2 (4) |  | 0.0 (5) | 7.7 (4) | 12.8 (4) |
| 4 | Ill-defined | 21 | 16 | 14 | | 20 | 0 |  | | | 14 | 0.0 (6) | 0.0 (6) | 79.5 (1) | 0.0 (6) |  | 0.0 (6) | 0.0 (6) | 9.4 (5) |
| 5 | Neonatal infections | 16 | 11 | 26 | | 31 | 25 |  | | | 10.7 | 1.7 (4) | 33.4 (1) | 0.0 (5) | 16.8 (3) |  | 35.3 (2) | 38.5 (1) | 17.1 (3) |
| 6 | Other | 0 | 0 | 0 | | 4 | 0 |  | | | 0 | 0.0 (5) | 0.0 (5) | 0.0 (6) | 0.0 (5) |  | 10.8 (4) | 4.3 (5) | 0.0 (6) |
|  | ***Agreement*** |  |  |  | |  |  |  | | |  | ***57*** | ***68*** | ***27*** | ***80*** |  | ***54*** | ***65*** | ***91*** |

* More obvious diagnoses. † Percentage of agreement between both physicians at initial stage of ICD coding, where both physicians assigned the same cause of death for the deceased record. The overall physician initial agreements for child and neonate age groups were 77.4% and 71.3%, respectively.
