# Supplementary material for: Automated versus physician assignment of cause of death for verbal autopsies: randomized trial of 9374 deaths in 117 villages in India
Source: BMC Med. 2019 Jun 27;17:116. doi: 10.1186/s12916-019-1353-2 (PMC6595581; doi:10.1186/s12916-019-1353-2)
Supplement: Supplementary file 13 — Comparison of population-level concordance in cause of death assignment for adults predicted between different algorithms for the automated assignment arm. (DOCX 19 kb) [file 12916_2019_1353_MOESM13_ESM.docx]

**Additional File 13: Comparison of population level concordance in cause of death assignment for adults predicted between different algorithms for the automated assignment arm**

| Comparator listed below | King-Lu | SmartVA | InSilicoVA | InSilicoVA-NT | InterVA-4 |
| --- | --- | --- | --- | --- | --- |
| NBC | 66 | 42 | 41 | 50 | 48 |
| King-Lu | * | 36 | 51 | 39 | 38 |
| SmartVA |  | * | 42 | 48 | 62 |
| InSilicoVA |  |  | * | 58 | 58 |
| InSilicoVA-NT |  |  |  | * | 76 |

Ages 12-69 years; n=4393, using data from all PHMRC sites as training data. The population level concordance between each algorithm and dual physician review was 46 for NBC, 43 for King-Lu, 65 for SmartVA, 64 for InSilicoVA, 69 for InSilicoVA-NT and 83 for InterVA-4. * Not applicable.
