# Supplementary material for: Automated versus physician assignment of cause of death for verbal autopsies: randomized trial of 9374 deaths in 117 villages in India
Source: BMC Med. 2019 Jun 27;17:116. doi: 10.1186/s12916-019-1353-2 (PMC6595581; doi:10.1186/s12916-019-1353-2)
Supplement: Supplementary file 14 — Cohen’s Kappa (confidence intervals) between algorithm predictions for adult automated assignment deaths (N = 4393). (DOCX 19 kb) [file 12916_2019_1353_MOESM14_ESM.docx]

**Additional File 14: Cohen’s Kappa (confidence intervals) between algorithm predictions for adult automated assignment deaths (N = 4393)**

| Comparator listed below | SmartVA | InSilicoVA | InSilicoVA-NT | InterVA-4 |
| --- | --- | --- | --- | --- |
| NBC | 0.17 (0.15, 0.18) | 0.18 (0.17, 0.20) | 0.12 (0.11, 0.14) | 0.13 (0.12, 0.15) |
| SmartVA | * | 0.17 (0.16, 0.19) | 0.33 (0.32, 0.35) | 0.35 (0.33, 0.36) |
| InSilicoVA |  | * | 0.16 (0.15, 0.18) | 0.20 (0.19, 0.21) |
| InSilicoVA-NT |  |  | * | 0.64 (0.63, 0.66) |

Average Kappa was 0.25 (standard deviation was 0.16) between the algorithms. King-Lu only produces population-level results, and thus, was not included. The Kappas between each algorithm and dual physician review were 0.16 (0.15, 0.18) for NBC, 0.42 (0.41, 0.44) for SmartVA, 0.26 (0.25, 0.28) for InSilicoVA, 0.47 (0.46, 0.49) for InSilicoVA-NT and 0.49 (0.47, 0.50) for InterVA-4. Due to small sample sizes for children and neonates, individual Cohen’s Kappa scores are not shown; however, the average Kappa for children and neonates were 0.23 (standard deviation of 0.16) and 0.13 (standard deviation of 0.18), respectively. * Not applicable
