# Supplementary material for: Automated versus physician assignment of cause of death for verbal autopsies: randomized trial of 9374 deaths in 117 villages in India
Source: BMC Med. 2019 Jun 27;17:116. doi: 10.1186/s12916-019-1353-2 (PMC6595581; doi:10.1186/s12916-019-1353-2)
Supplement: Supplementary file 15 — Percent population-level concordance in cause of death distribution between automated assignment and standard (physician assignment) verbal autopsies, by algorithms and age groups using all results, including pilot site (1215 additional deaths). (DOCX 20 kb) [file 12916_2019_1353_MOESM15_ESM.docx]

**Additional File 15: Percent population level concordance in cause of death distribution between automated assignment and standard (physician assignment) verbal autopsies, by algorithms and age groups using all results, including pilot site (1215 additional deaths)**

|  | Mean (SD) | *Require training data* | | | |  | *Do not require training data* | |
| --- | --- | --- | --- | --- | --- | --- | --- | --- |
| Age Group |  | NBC | King-Lu | SmartVA | InSilicoVA |  | InSilicoVA-NT | InterVA-4 |
| **Adult** | 62 (15) | 48 | 45 | 57 | 66 |  | 77 | 80 |
| **Child** | 57 (13) | 50 | 54 | 36 | 66 |  | 66 | 68 |
| **Neonate** | 59 (17) | 57 | 65 | 29 | 78 |  | 54 | 68 |

Mean and standard deviation (SD) of the population level concordance attained for the automated assignment VA algorithms when using data from all PHMRC sites as the training data, stratifying by age groups: adult (12-69 years); child (28 days – 11 years); and neonate (0-27 days). The algorithms were run on automated assignment data, including pilot study automated assignment deaths (total of 9,809 adults, 469 child and 311 neonate deaths). InSilicoVA-NT and InterVA-4 do not require training data, whereas SmartVA was pre-trained using data from all PHMRC sites.
