# Supplementary material for: Automated versus physician assignment of cause of death for verbal autopsies: randomized trial of 9374 deaths in 117 villages in India
Source: BMC Med. 2019 Jun 27;17:116. doi: 10.1186/s12916-019-1353-2 (PMC6595581; doi:10.1186/s12916-019-1353-2)
Supplement: Supplementary file 16 — Percent of deaths by cause from the standard (physician assignment) arm compared to deaths (2010–2013) in the Indian Million Deaths Study in the trial state by age groups. (DOCX 286 kb) [file 12916_2019_1353_MOESM16_ESM.docx]

**Additional File 16: Percent of deaths by cause from the standard (physician assignment) arm compared to deaths (2010-13) in the Indian Million Deaths Study in the trial state by age groups**


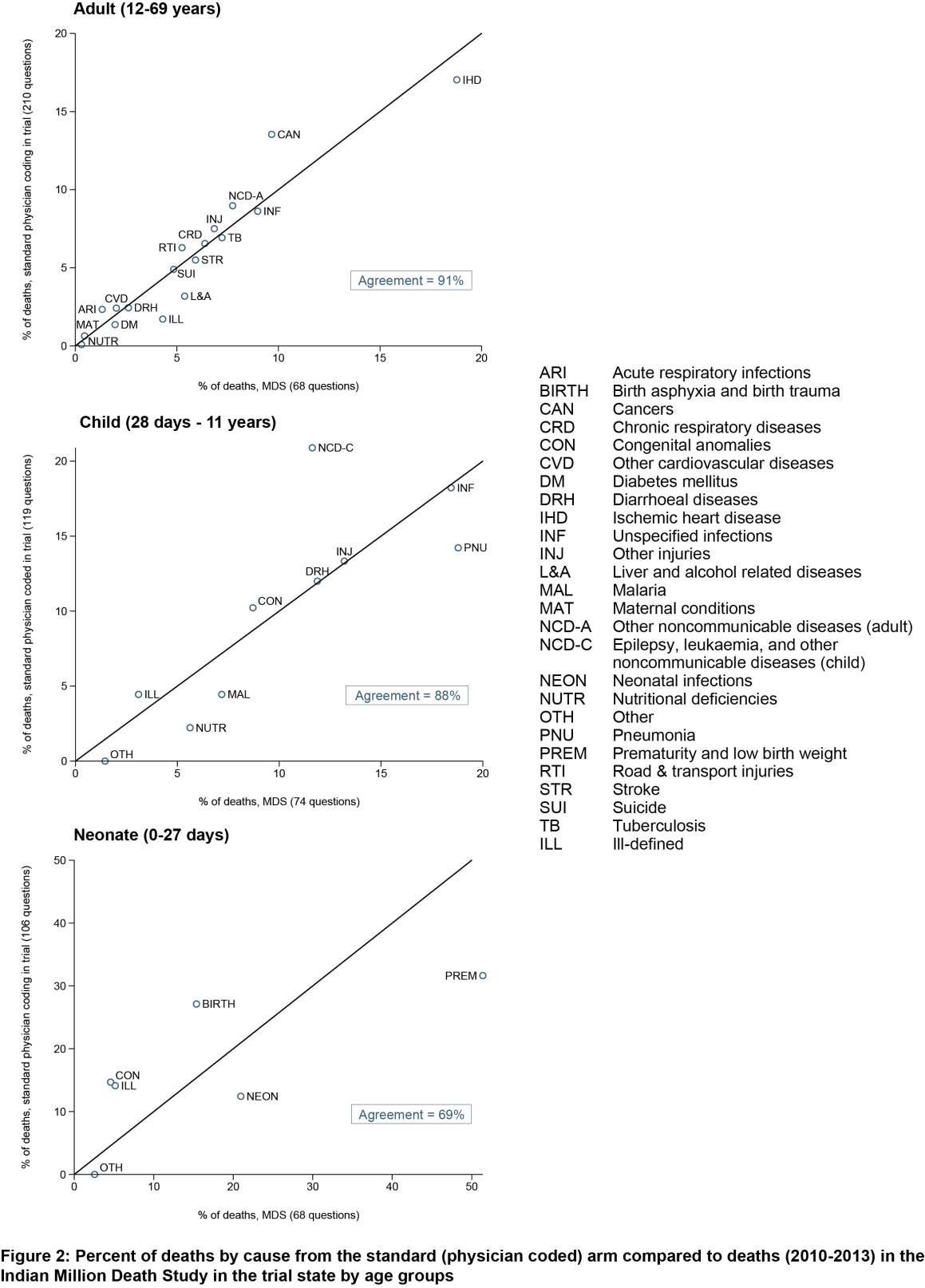


Rural Million Death Study (MDS) deaths include deaths from the three Indian states where data collection occurred for this study, including the pilot site, Maharashtra. Results excluding the latter site were nearly identical. MDS deaths were weighted by sampling probability: adult (9,841 MDS deaths); child (1,019 MDS deaths); and neonate (1,328 MDS deaths). These MDS deaths are compared to the physician coded study group by age: adult (4,838 physician assigned trial deaths); child (225 physician assigned trial deaths); and neonate (177 physician assigned trial deaths). The agreement in cause of death distributions between the physician coded study group and weighted MDS deaths described above for adult, child, and neonate were 91%, 88%, and 69%, respectively.
