# Supplementary material for: Automated versus physician assignment of cause of death for verbal autopsies: randomized trial of 9374 deaths in 117 villages in India
Source: BMC Med. 2019 Jun 27;17:116. doi: 10.1186/s12916-019-1353-2 (PMC6595581; doi:10.1186/s12916-019-1353-2)
Supplement: Supplementary file 17 — Percent of deaths by cause for adults (12–69 years) and children (0–4 years) in the Indian Million Death Study (2001–2013) compared to deaths in the 3% Million Death Study resample. (DOCX 254 kb) [file 12916_2019_1353_MOESM17_ESM.docx]

**Additional File 17: Percent of deaths by cause for adults (12-69 years) and children (0-4 years) in the Indian Million Death Study (2001-2013) compared to deaths in the 3% Million Death Study resample**


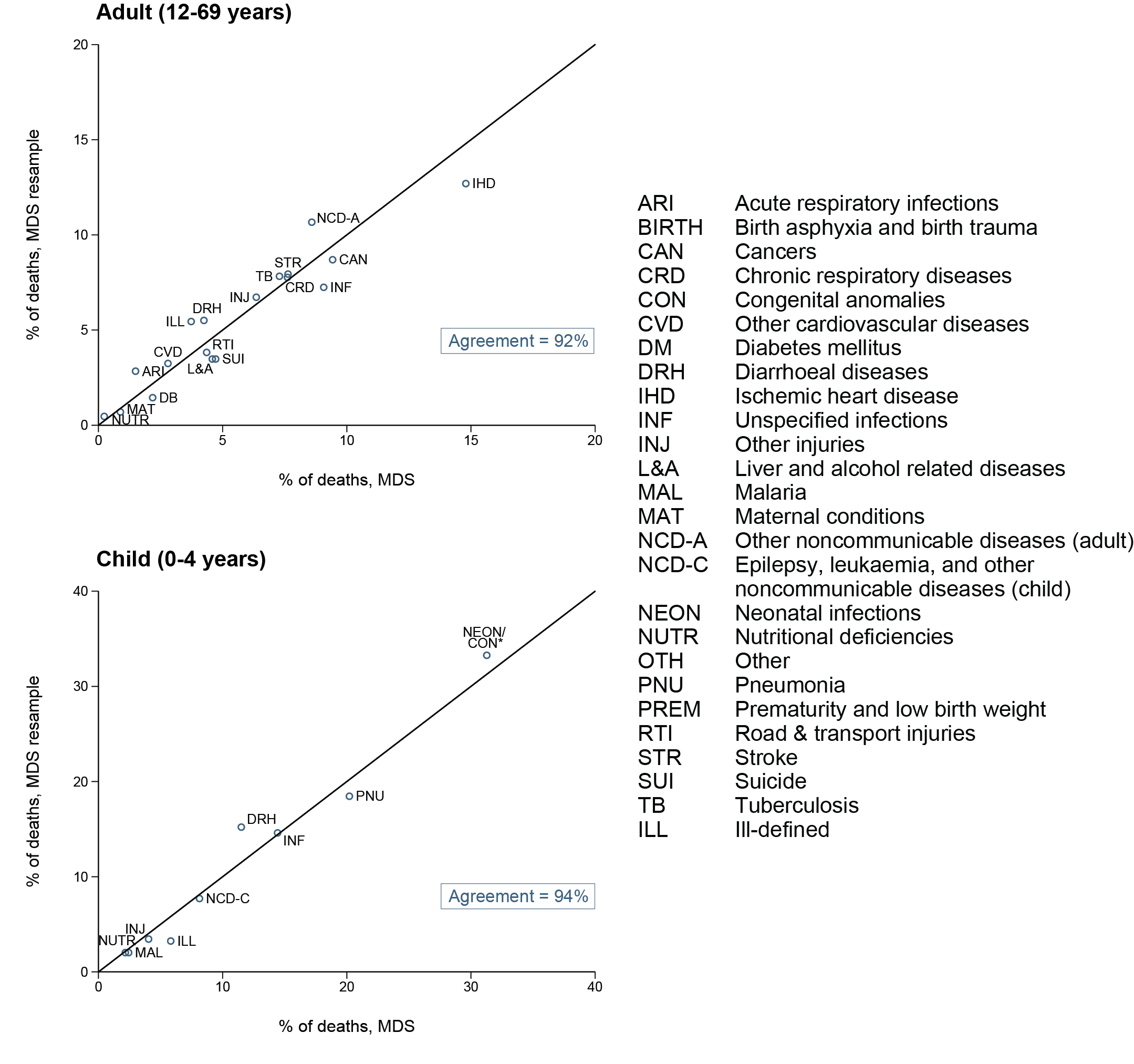


Million Death Study (MDS) deaths were weighted and grouped by age (286,790 adult deaths; 88,402 child deaths). These weighted MDS deaths are compared to the 3% MDS resample, consisting of independently surveyed deaths from the same survey framework as the original MDS study (1,725 adult deaths; 493 child deaths). ICD-10 cause of death codes from the MDS were grouped into broad categories by age group. The agreement in cause of death distributions between the MDS deaths and MDS resample deaths described above for adult and child were 92% and 94%, respectively.
