# Supplementary material for: Automated versus physician assignment of cause of death for verbal autopsies: randomized trial of 9374 deaths in 117 villages in India
Source: BMC Med. 2019 Jun 27;17:116. doi: 10.1186/s12916-019-1353-2 (PMC6595581; doi:10.1186/s12916-019-1353-2)
Supplement: Supplementary file 18 — Percent of sub-study deaths by cause from lay surveyor versus physician collected adult verbal autopsies. (DOCX 188 kb) [file 12916_2019_1353_MOESM18_ESM.docx]

**Additional File 18: Percent of sub-study deaths by cause from lay surveyor versus physician collected adult verbal autopsies**


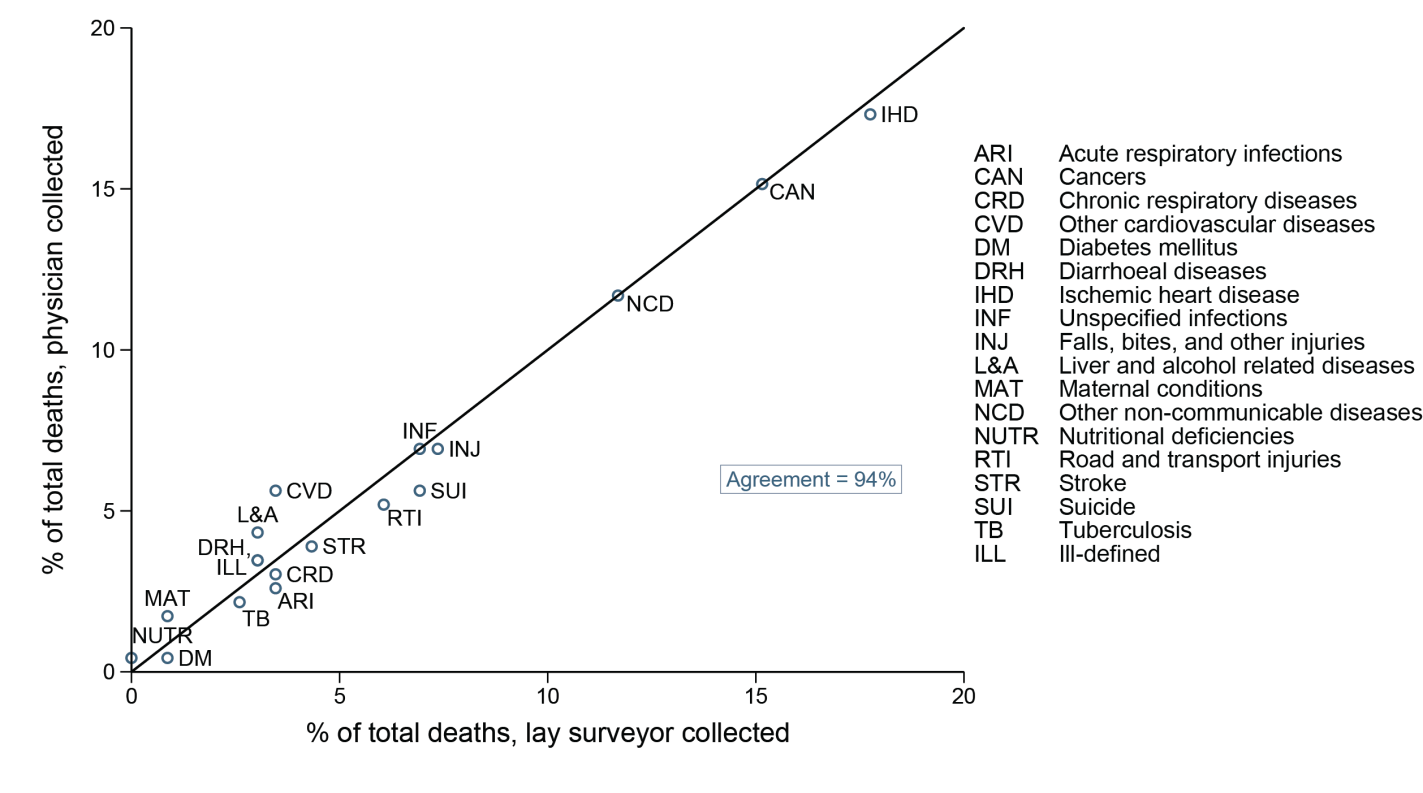


231 adult (12-69 years) deaths were randomly selected in the Amravati district, and verbal autopsies were collected independently by lay surveyors and physicians for the same deaths. ICD-10 causes of death were assigned to the collected verbal autopsies by physicians, who were not involved in the data collection, and are summarized here in 18 broad cause of death categories and grouped by data collector. The cause of death agreement between these data collection groups is 94%.
