# Supplementary material for: Automated versus physician assignment of cause of death for verbal autopsies: randomized trial of 9374 deaths in 117 villages in India
Source: BMC Med. 2019 Jun 27;17:116. doi: 10.1186/s12916-019-1353-2 (PMC6595581; doi:10.1186/s12916-019-1353-2)
Supplement: Supplementary file 19 — Original study protocol. (DOCX 21 kb) [file 12916_2019_1353_MOESM19_ESM.docx]

**Additional File 19: Original Study Protocol**

| **Original Study Protocol**  **A direct comparison of physician versus computerized algorithm coding for verbal autopsies in populations without medical attention at the time of death**    **Centre for Global Health Research (CGHR)**  **St. John’s Research institute (SJRI),**  **Opp. BDA Complex, Koramangala,**  **Bengaluru - 560034**  **Version 2, April 12,**  **Correspondence:**  **Dr. Suresh Rathi**  **Cell No: +91-9945908671**  **Email: rathis@smh.ca** |
| --- |

ABBREVIATION:

AP Andhra Pradesh

CGHR Centre for Global Health Research m

CO Coordinating Office

E-RHIME Electronic Routine, Representative, Re-sampled, Household Interview of Mortality with Medical Evaluation

EVA Electronic Verbal Autopsy

FO Field Officer

GPS Global Positioning System

GU Gujarat

ICD-10 International Classification of Diseases – 10

IHME Institute of Health Matrix and Evaluation

KN Karnataka

LMIC Low and Middle Income Countries

MDS Million Death Study

MH Maharashtra

NNT Non-Narrative Tool

PI Principal Investigator

SRS Sample Registration System

RHIME Routine, Representative, Re-sampled, Household Interview of Mortality with Medical Evaluation

UP Utter Pradesh

VA Verbal Autopsy

WHO World Health Organization
